# Marine resource abundance drove pre-agricultural population increase in Stone Age Scandinavia

**DOI:** 10.1038/s41467-020-15621-1

**Published:** 2020-04-24

**Authors:** J. P. Lewis, D. B. Ryves, P. Rasmussen, J. Olsen, L. G. van der Sluis, P. J. Reimer, K.-.L. Knudsen, S. McGowan, N. J. Anderson, S. Juggins

**Affiliations:** 1 Geography and Environment, Loughborough University, Loughborough, LE11 3TU UK; 2 Environmental Archaeology and Materials Science, National Museum of Denmark, Brede Værk, I.C. Modewegsvej, DK-2800 Kgs. Lyngby, Denmark; 3 Department of Physics and Astronomy, Aarhus University, Ny Munkegade 120, DK-8000 Aarhus C, Denmark; 4 School of Natural and Built Environment, Queen’s University Belfast, Belfast, BT7 1NN UK; 5 Department of Earth Science, Aarhus University, Høegh-Guldbergs Gade 2, DK-8000 Aarhus C, Denmark; 6 School of Geography, University of Nottingham, Nottingham, NG7 2RG UK; 7 School of Geography, Politics and Sociology, Newcastle University, Newcastle upon Tyne, NE1 7RU UK

**Keywords:** Palaeoecology, Palaeoclimate, Stable isotope analysis, Archaeology

## Abstract

How climate and ecology affect key cultural transformations remains debated in the context of long-term socio-cultural development because of spatially and temporally disjunct climate and archaeological records. The introduction of agriculture triggered a major population increase across Europe. However, in Southern Scandinavia it was preceded by ~500 years of sustained population growth. Here we show that this growth was driven by long-term enhanced marine production conditioned by the Holocene Thermal Maximum, a time of elevated temperature, sea level and salinity across coastal waters. We identify two periods of increased marine production across trophic levels (P1 7600–7100 and P2 6400–5900 cal. yr BP) that coincide with markedly increased mollusc collection and accumulation of shell middens, indicating greater marine resource availability. Between ~7600–5900 BP, intense exploitation of a warmer, more productive marine environment by Mesolithic hunter-gatherers drove cultural development, including maritime technological innovation, and from ca. 6400–5900 BP, underpinned a ~four-fold human population growth.

## Abstract (web summary)

How the development of human societies is influenced through their ecological environment and climatic conditions has been the subject of intensive debate. Here, the authors present multi-proxy data from southern Scandinavia which suggests that pre-agricultural population growth there was likely influenced by enhanced marine production.

## Introduction

Climate and environmental change ranks among the strongest and possibly also the most frequent triggers of cultural change and innovation^1,2^. Whilst research has focussed on the consequences of cultural change, antecedent periods to major cultural transformations are critical times for contextualising past human behaviour, understanding adaptation and resilience to (internal and external) resource pressures and the processes that underpin how such transitions operate. Human response to changing resource availability is a crucial factor driving cultural and demographic trajectory^3,4^. Adaptation to, and exploitation of, new resource opportunities have driven pre-industrial population patterns in complex relationships with environmental change, demographic and cultural factors^1,2,5^, yet understanding past human-environment interactions still remains a grand challenge for archaeology^6^. The introduction of agriculture brought about one of the greatest cultural and demographic changes in world history enabling a population boom at the onset of the Neolithic across Europe^1,7^. However, in Southern Scandinavia the introduction of agriculture was delayed (compared to contiguous parts of Germany) by several centuries^7,8^, with human subsistence being largely dependent on marine protein including marine mammals, fish, birds and molluscs^9–13^. Chronologically robust, high resolution and high quality datasets from prehistory can provide key insights to debates on contemporary global environmental change and its potential cultural implications^12,14–16^.

Holocene population trends across the world have been extensively studied with the development of ^14^C dates as data methods^3,4,7,17,18^. This approach has shown the global population increase to be very gradual until recently, with near to zero population growth (0.04%) throughout much of history^18,19^, though punctuated by distinct boom and bust phases^7^, with catastrophic periods identified as a key factor (along with altered mean vital rates) constraining population growth^18^. The repeatable, but regionally variable boom and bust pattern has generated considerable debate with regard to the possible drivers of human population responses to a multitude of factors and stressors, including, socio-economic status, disease, war, environmental and climatic change as well as resource availability^3,14,20–22^. In northern Europe, the boom and bust pattern of agrarian societies is well known^3,7^, but population changes in pre-agricultural hunter-gatherer communities are less well understood. Some recent studies have identified rapid pre-agricultural population shifts, linked to the abundance of key natural (both terrestrial and aquatic) resources, and likely driven by environmental and/or climatic conditions^4,23^. Enhanced use of marine resources has been widely observed in pre-agricultural societies across northwest Europe^4,10,24,25^ though away from higher latitudes (above agricultural limits; 58–60°N), their potential importance in driving both population and cultural changes remains poorly understood. However, recent geoarchaeological studies have inferred that, in conjunction with terrestrial and freshwater resources, marine resources might have contributed to the pre-agricultural population increases in the eastern/central Baltic^4,23^.

It has been shown that hunter-gatherer populations can increase rapidly when resources are abundant and environmental conditions are favourable^17,21,25^, but classically hunter-gatherer communities have been assumed to exploit marine and aquatic resources only when more cost-benefit (i.e. terrestrial) resources are unavailable^26,27^. For example, in the tropics and lower latitudes subsistence is predominately terrestrial (plant-based), whilst in general greater dependence is placed on non-plant resources (particularly terrestrial animal protein) with distance from the equator^26,27^. Where plant-resources are abundant and/or hunting of terrestrial animals favourable, exploitation of aquatic (including marine) resources, if available, were likely only exploited as supplements to the diet (<25% of total diet^27^). Binford^26^ suggested that humans only turn to aquatic resources when terrestrial foods are unavailable, such as in cold, high latitudes (e.g. boreal forests and/or smaller islands), where primary production is lower and high quality terrestrial hunting grounds, necessary to sustain large or increasing populations, are unavailable. For example, marine resources became increasingly important along the Pacific coast of northwest America throughout the Holocene, likely due to habitat deterioration inland (e.g. transition from open woodland to closed boreal forest during the Holocene Thermal maximum^28^), reduction in terrestrial game hunting populations and presence of rich marine environment yielding alternative resources^29,30^. After ca. 5000 calibrated years before present (AD 1950; hereafter BP) in the Pacific northwest, there is evidence for further intensified use of marine resources, particularly salmon and shellfish (including emergence of large shell middens) as sea levels stabilised and substrates suitable for seaweed and mollusc habitation developed^29–31^.

If, as is generally assumed, subsistence resources are selected on a cost-benefit basis, the Southern Scandinavian case represents an interesting enigma, as the early Holocene Maglemose (ca. 11,600–8400 BP) culture of Southern Scandinavia appear to have subsisted on a mixed terrestrial/freshwater diet, but both the succeeding Kongemose (ca. 8400–7400 BP) and Ertebølle (ca. 7400–5900 BP) cultures predominately utilised marine resources^9,10^. For some reason the Kongemose and Ertebølle cultures chose marine resources over terrestrial/freshwater resources, including over agriculturally-derived protein for several hundred years (ca. 6600-5900 BP), before the sudden (possibly traumatic^32^) introduction of agriculture at ca. 5900 BP. This sequence suggests that between ca. 8400 and 5900 BP either, more cost-effective (terrestrial) resources were in short supply, or that marine resources were more cost-effective than terrestrial plants and animals (and more cost-effective than agriculture between ca. 6600 and 5900 BP).

**Fig. 1:**
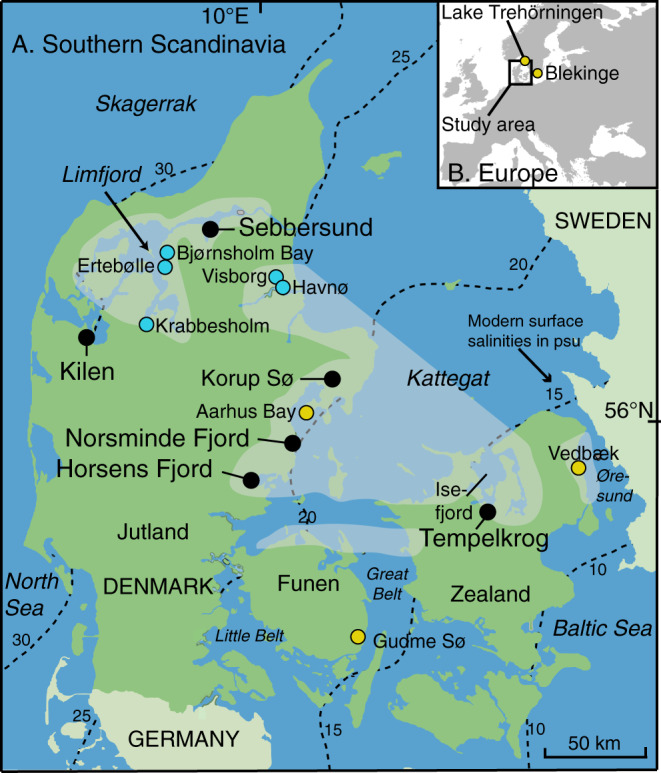
Map of Denmark with location of the study sites (black dots). Shading indicates the distribution of middle and late Stone Age shell middens across >7300 km of coastline with key middens indicated (blue dots). Other important sites mentioned in the text are shown with yellow dots. Salinity contour lines based on Dahl et al.^64^.

As is apparent from above, a central question in the population-resource nexus^3,4,6^ is: did Southern Scandinavian cultures select marine resources due to their abundance at this time (i.e. increased marine production), and did this subsequently enable population growth, or were hunter-gatherers forced to use more marine resources, due to population packing, driven by increased terrestrial production during the Holocene Thermal Maximum (HTM; ca. 8000–4000 BP)? To address this question, we use a unique multiproxy dataset combining regional climate, sea-level, archaeological and environmental data from six coastal sites across Denmark (Fig. 1). We show that high marine production coincides with higher temperatures, higher sea level and higher seawater salinity at the peak of the HTM (ca. 7500–6000 BP). This led to an expansion of marine resource utilisation by Mesolithic hunter-gatherers in Southern Scandinavia and in turn coincided with a substantial increase in population in the centuries immediately preceding the introduction of agriculture^7^.

## Results

### Climate and marine environmental change

**Fig. 2:**
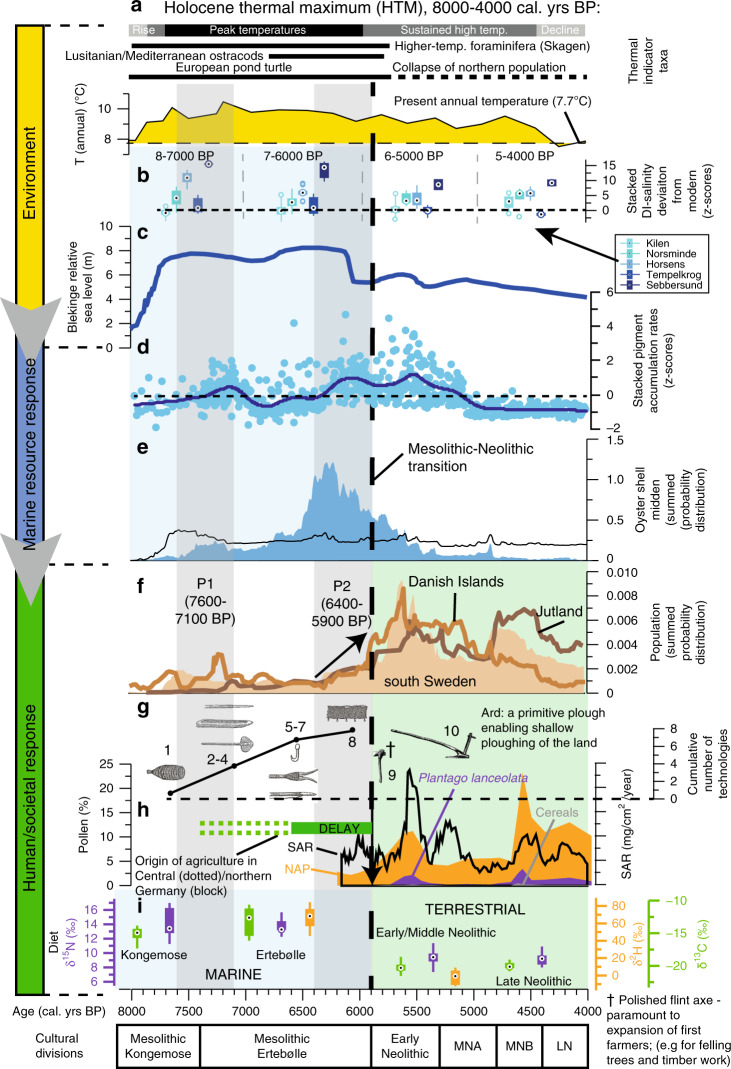
Southern Scandinavian regional climate, sea level and coastal environment during the Holocene thermal maximum and human response. Environmental variables: **a** Pollen-inferred mean annual air temperature based on a sediment succession from Lake Trehörningen^37^, southwest Sweden and examples of warmth-demanding indicator species present during the Holocene thermal maximum (HTM), but absent/rare in Danish waters today (see Supplementary Fig. 21). **b** Diatom-inferred salinity expressed as deviations from modern salinity for five Danish coastal sites. Order of box plots from left to right: Kilen, Horsens Fjord, Norsminde Fjord, Tempelkrog, Sebbersund (repeats for each time period). **c** Sea level, Blekinge, southeast Sweden^33^. Ecological and human response variables: **d** Total sedimentary pigment accumulation rate z-scores with lowess smoother (span 0.1) for three Danish coastal sites (Kilen, Horsens Fjord and Tempelkrog). P1 and P2 refer to periods of pre-agricultural marine production increase across Southern Scandinavia. **e** Summed probability distribution (SPD) of ^14^C-dates on shells of the European flat oyster (*Ostrea edulis*) from Danish shell middens as a proxy for total midden abundance and marine resource availability (see text and Supplementary Note 2). Black line indicates expected probability distribution. **f** Population density proxy for southern Sweden, Jutland and the Danish islands during the mid-Holocene^7^. **g** Cumulative number of technologies used to exploit the marine environment over the study period^44, 65^ and agricultural technology post 5900 BP. 1. Fish trap; 2–4. Lance, dugout canoe, paddle; 5–7. Fish hook, leicester, paddle; 8. Fish net; 9. Polished flint axe; 10. Ard. (see Supplementary Table 5). **h** Land-use/agricultural change indicators: sediment accumulation rate (SAR) and percentage of non-arboreal pollen (NAP), *Plantago lanceolata* (ribwort plantain) and cereal pollen at Lake Gudme Sø^48^, Funen, Denmark. **i** Isotopic analyses of δ^13^C, δ^15^N and δ^2^H data from Mesolithic and Neolithic humans and dogs showing shift from a predominantly marine (more positive values for all isotopes) to terrestrial diet (more negative values)^10, 47^. All box plots (in **b**, **i**) show maximum, minimum, interquartile range and median. Cultural divisions after Fischer and Kristensen^8^; MNA, Middle Neolithic A; MNB, Middle Neolithic B; LN, Late Neolithic.

**Fig. 3:**
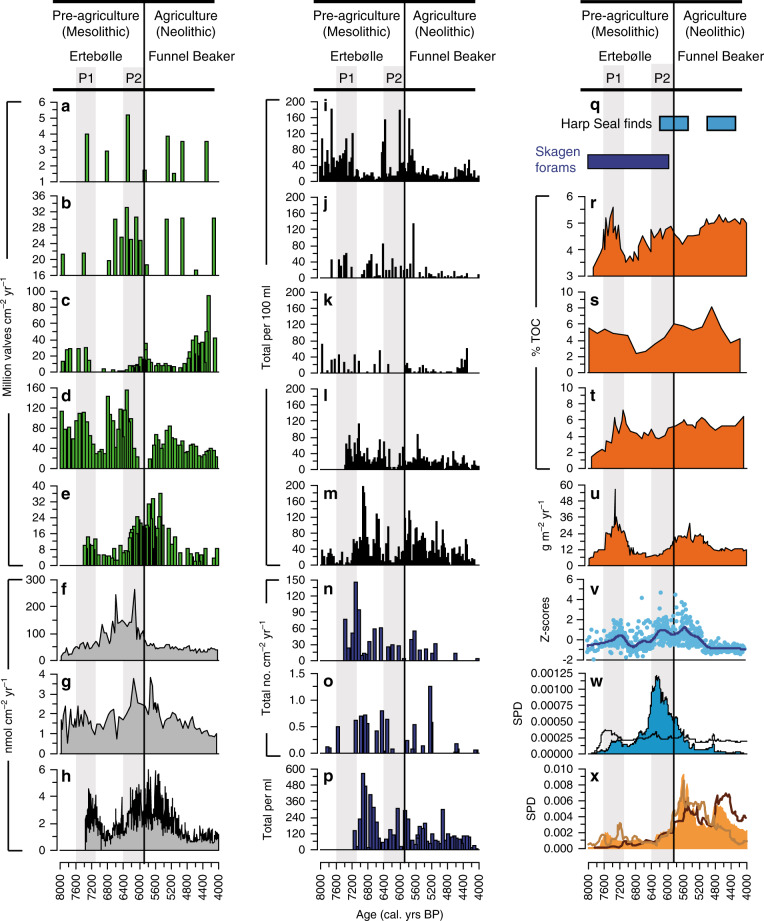
Marine productivity indicators across Southern Scandinavia and the Baltic Sea from 8000–4000 BP. P1 and P2 refer to periods of pre-agricultural marine production increase across Southern Scandinavia. **a**–**e** Diatom flux (**a** Sebbersund, **b** Tempelkrog; **c** Norsminde Fjord; **d** Horsens Fjord; **e** Kilen). **f**–**h** Total sedimentary pigment flux: (**f** Tempelkrog; **g** Horsens Fjord; **h** Kilen). **i**–**m** Number of molluscs per 100 ml of wet sediment (**i** Tempelkrog; **j** Sebbersund; **k** Korup Sø; **l** Kilen; **m** Horsens Fjord). **n**–**p** Foraminiferal flux (**n** Kilen; **o** Norsminde Fjord; **p** Horsens Fjord). **q** Faunal indicators of high productivity: appearance of ^14^C dated harp seal (*Phoca groenlandica*) remains found in Denmark^41^ and phase of increased flux of foraminifera present in the northern Kattegat^66^. **r**–**u** Organic carbon indicators of productivity from the Baltic Sea. Percentage total organic carbon (TOC) in sediment sequences from **r** Little Belt^67^, **s** Gotland Basin^68^, **t** Gotland Basin^69^, **u** TOC flux at Gotland Basin^70^. **v**–**x** Key summary data from this study for comparison (see text and Fig. 2 for details); **v** Total sedimentary pigment flux z-scores (with lowess smoother; span 0.1; based on the data plotted in **f**–**h**). **w** Summed probability distribution (SPD) of ^14^C-dates of shells of the European flat oyster (*Ostrea edulis*) from Danish shell middens. Black line indicates expected probability distribution**. x** Population density proxy for southern Sweden, Jutland and the Danish islands during the mid-Holocene^7^.

The landscape of Southern Scandinavia was completely reconfigured during the Early Holocene (pre ca. 7600 BP) by rising sea levels^33–35^ which flooded huge tracts of land, including the land bridge between Denmark and Sweden, and thus turning large parts of Denmark into a series of islands. At the same time increasing temperatures associated with the onset of the Holocene Thermal Maximum^36,37^ (Fig. 2a) drove terrestrial vegetation development, with open ground vegetation and pioneer tree taxa being replaced by dense mixed deciduous woodland^38^. The succeding HTM lasted ~4000 years (ca. 8000–4000 BP) in Southern Scandinavia yielding mean annual temperatures ~2.5 °C^37^ higher during its peak (ca. 7500–6000 BP) than the recent pre-industrial period^36,37^, whilst peak sea levels are recorded across Southern Scandinavia between 7600 and 5000 BP^33–35^. Diatom-inferred salinity values^39,40^ from five Danish fjord sites (Figs. 1 and 2b; Supplementary Note 1) show that coastal salinity across the HTM was higher than today, in keeping with higher regional sea level^33,35^ (Fig. 2c). A range of thermophilic (aquatic and terrestrial) and/or high salinity taxa were also present during the peak of the HTM (Figs. 2a and 3q; Supplementary Note 3; Supplementary Fig. 22) that are extinct or rare today in Southern Scandinavia, though some are reappearing with present-day global warming^12^.

Across the peak of the HTM (ca. 7500–6000 BP) and period of highest sea levels (ca. 7600–5000 BP), sedimentary pigment biomarkers reveal high algal production in the late Mesolithic and into the early Neolithic (Fig. 2d). We show here that superimposed upon this general high marine production, there are two distinct phases of elevated marine production between ca. 7600 and 7100 BP (labelled P1; Fig. 2) and ca. 6400–5900 BP (P2, Fig. 2) that coincide with key changes in the archaeological record (i.e. human response; outlined below). Both Kilen and Horsens Fjord show some evidence for increased marine algal production between ca. 7600 and 7100 BP (P1, Fig. 3g, h), and whilst there is clearly some variability amongst some other sites for some proxies across trophic levels (Fig. 3), the composite pigment accumulation rate (based on Z-scores; Fig. 2d) supports a phase of increased algal production between ca. 7600 and 7100 BP. However, the second, and larger, event (P2, ca. 6400–5900 BP), has greater regional coherence (Figs. 2d and 3), with all three sites with sediment pigment data available (Tempelkrog, Horsens Fjord and Kilen; Fig. 3), showing clear evidence for elevated marine production between ca. 6400 and 5900 BP. These three sites also show good agreement between highest algal, foraminiferal and marine mollusc abundance (Fig. 3a–p), and document extended phases of high primary and/or secondary production between ca. 6400 and 5900 BP (Fig. 2d), indicating that the mid-Holocene was generally characterised by high marine productivity across trophic levels. In a number of proxies high productivity extends up until ca. 5000 BP, but changes between ca. 5900 and 5000 post-date the introduction of agriculture and are likely to be driven (at least partly) by human impact on the catchment (see below; Fig. 2h).

Few other studies have directly inferred marine palaeoproductivity in the Kattegat/Baltic Sea, though several have alluded to high productivity between ca. 8000 and 5000 BP (e.g. TOC data, Fig. 3r–u), initially following sea-level rise and increased exchange of oceanic water, and later around the late Mesolithic/Early Neolithic. A peak of harp seal finds in the Belt Sea/Kattegat area between ca. 6200 and 5500 BP (focussed around 5900 BP; Fig. 3q) also suggests higher biological productivity and increased inflow of highly saline water from the North Sea during the Late Mesolithic/Early Neolithic period^41^. Whilst some of the multiple records and diverse coastal proxies shown here (Fig. 3) document increased marine productivity across the P1 and P2 events, there is a general trend of high marine productivity spanning from ca. 7600 to 5000 BP. This coincides with highest sea levels^33^ and the peak temperatures of the HTM (ca. 7500–6000 BP based on the vast majority of data for Southern Scandinavia^36,37^ (Fig. 2a), though in contrast to data by Warden et al.^42^ from the remote Gotland Basin; Supplementary Note 3).

### Human response to environmental change

Human response is documented by archaeological records of shell midden presence/duration, frequency and composition, population density, technological advancement and human diet (Fig. 2e–i). We apply a novel shell midden accumulation indicator based on 231 calibrated ^14^C dates of oyster shells (using the summed probability distribution method, SPD) collected from Danish shell middens as a proxy for human coastal marine utilisation, and by implication, marine resource availability. Shell middens first appear around 7600 BP in Denmark^13^, and steadily increase in frequency (and volume) throughout the late Mesolithic and into the Early Neolithic, reaching a maximum ca. 6400–5700 BP (Fig. 2e). Active middens were continually present along Danish coasts until ca. 4200 BP, after which their presence became much more sporadic before disappearing completely in the Bronze Age^13^.

The European flat oyster (*Ostrea edulis*) has a very scattered distribution in Danish waters today. It appears in the earliest Danish shell middens ca. 7600 BP coinciding with a rise in temperature, sea level and salinity^34,35^ associated with the peak of the HTM (ca. 7500–6000 BP)^36,37^ (see Supplementary Note 3). The oyster was quickly exploited, and often dominates coastal shell middens several metres thick, though other molluscs and vertebrates are also plentiful^13^. Post sea-level rise, terrestrial sediment in-wash from pre-agricultural landscapes remains low in coastal sites (e.g. Supplementary Figs. 1 and 21) with less runoff under warmer and relatively dry climatic conditions, providing a relatively hard bottom substrate suitable for oysters, which were able to consume increasing autochthonous primary production, possibly supported by diffuse nutrient mobilisation from inundated coastal margins due to higher sea level (Fig. 2c). Our oyster-derived shell midden abundance curve (Fig. 2e) is a surrogate for intense marine resource exploitation between ca. 7600 and 5500 BP; relatively non-nutritious shell fish are unlikely to have represented major staple resources but may have been important to fill seasonal gaps in resource availability^43^. A large number and diversity of vertebrates are present in the shell middens and other coastal archaeological sites, including fish (both marine and freshwater), bird and mammal remains from marine, freshwater and terrestrial habitats, that attest to a broad and intense exploitation of aquatic resources^11,12,44,45^ (see Supplementary Note 2).

We show here that periods of peak shell midden accumulation (Fig. 2e) occur broadly synchronous with phases of high marine production (P1 and P2; Fig. 2). Perhaps unexpectedly, marine resource exploitation (as indicated by shell midden abundance; Fig. 2e) appears to peak before marine primary productivity during both P1 and P2 (Fig. 2). This might be an artefact of the chronological models resulting from ^14^C dating uncertainties (~200 years) between the different records, or (at least in part) a real offset related to ecological and/or human impact controls on marine resources. For example, it is possible that the environmental carrying capacity of organisms at higher trophic levels (consumed by humans) might be reached before algal production peaks. Alternatively, intensive marine resource exploitation following sustained human population growth might have reduced populations of key marine resources (e.g. oysters) prior to peak marine primary production, perhaps even creating a positive feedback loop by reducing grazing pressure placed on primary producers. However, it is not possible to decide between such competing hypotheses within the current dataset.

As marine ecosystem production progressively developed, and prior to the introduction of agriculture in this region at ca. 5900 BP, there was a significant population increase across southern Sweden, Jutland and the Danish Isles (Supplementary Table 4; Supplementary Fig. 20), especially in the last 500 years of the Mesolithic^7^ (~4-fold; in Fig. 2f). The first population increase (ca. 7600–7100 BP) occurs broadly synchronously with climate warming and a rise in marine productivity during the early HTM (coincidental with marine production increase P1; Fig. 2d). This would underpin greater production in the coastal ecosystem and, in turn, a pulse of marine resources including shellfish (shell-midden accumulation; Fig. 2e) and human population (Fig. 2f) exploiting these resources in response. However, the second, and larger, event (ca. 6400–5900 BP) shows a clear population increase at a time of major rise in marine production (P2; Figs. 2d and 3) and coastal resources during the period of most intensive marine resource exploitation (Fig. 2e–i). This population increase clearly started prior to the introduction of agriculture (Fig. 2f), in contrast to the early Neolithic population increase that reflects culturally driven changes (including migration^46^) associated with the onset of arable and pastoral farming. Maritime adaptations and technological innovation to changing resource availability supported the pre-agricultural population rise across Southern Scandinavia^7^ (Fig. 2g). In addition to new tools and pottery^13^, hunter-gatherers developed multiple innovative technologies to exploit marine ecosystems over the late Mesolithic^44^ (Supplementary Table 5).

Isotopic analyses of human remains clearly show the importance of marine protein during the late Mesolithic (Fig. 2i), with a shift to a marine-based diet occurring at the boundary between the Maglemose and Kongemose culture^10^ (ca. 8400 BP), with a (non-agricultural) terrestrial diet predominating in the Maglemose period. This shift, based on the available data, broadly coincides with substantial sea-level rise and the transition to higher salinity, accessible fjord waters dominating Southern Scandinavian coasts^10,33,35^. Throughout the Kongemose and Ertebølle period there is a strong marine signature (δ^13^C = –13‰, δ^15^N = 13‰,) before a fundamental shift to largely terrestrial subsistence in the Neolithic^9,10,47^ (δ^13^C = –20‰, δ^15^N = 9.5‰; Supplementary Dataset 1). In addition to the commonly used δ^13^C and δ^15^N ratios, δ^2^H ratios (from human skeletons from the coastal Limfjord) function as an additional trophic level indicator^47^, and provide new evidence that support this interpretation, with values shifting from +66‰ (Mesolithic) to –5‰ (Neolithic) (Fig. 2i).

### Links between land and sea from the early Neolithic

**Fig. 4:**
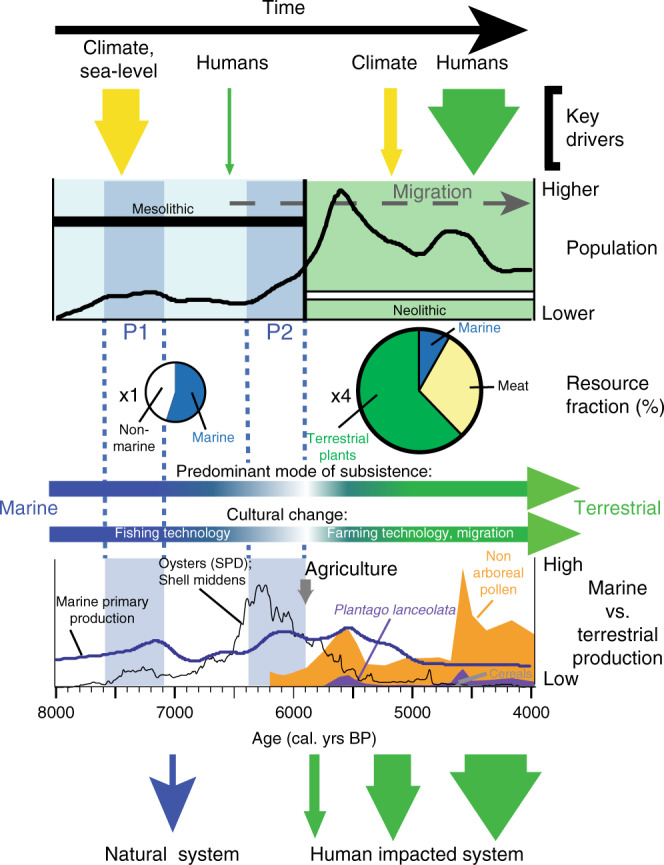
Conceptual model illustrating the complex population-culture-environment interrelationships in Southern Scandinavia during the late Mesolithic and early Neolithic (the Holocene thermal maximum). Relative size of Mesolithic and Neolithic populations are based on the four-fold difference in population in period P2 (ca. 6400–5900 BP; i.e. x1) and Neolithic (ca. 5900–4000 BP; i.e. x4) from the three Scandinavian population curves^7^ (Fig. 2f). Resource fraction represents the contributions of marine and terrestrial food sources to diet based on stable isotope measurements (δ^13^C, δ^15^N and δ^2^H) on Danish archaeological remains^10, 47^; *n* = 14 for Kongemose, *n* = 12 for Ertebølle, *n* = 42 for earlier Neolithic (ca. 5900–5000 BP) and *n* = 18 for later Neolithic (ca. 5000–4000 BP). For hydrogen *n* = 4 for Mesolithic and *n* = 8 for Neolithic. Anthropogenic impact on terrestrial (and coastal) systems increasingly supplants natural (e.g. climatic) variability as the dominant driver of ecosystem change under technological, socio-cultural (including migration) and demographic development in the Neolithic as reliance on marine resources diminishes.

With the introduction of agriculture in Southern Scandinavia ca. 5900 BP, the landscape witnessed major forest clearance, which led to large-scale changes in sediment and nutrient transfer from terrestrial to aquatic systems (Fig. 2h). During the late Mesolithic, the dense vegetation cover and low disturbance regime resulted in only sparse erosional inputs to lakes, watercourses and coasts. Widespread land clearance during the Neolithic is shown by pollen indicators of arable (cereals) and pastoral farming (ribwort plantain; *Plantago lanceolata;* Figs. 2h and 4) and coeval sedimentary evidence for elevated erosional soil export and nutrient transfer to freshwater bodies^48,49^. This ultimately impacted coastal areas and such enhanced nutrient export may explain why marine production remained high in the early Neolithic until ca. 5000 BP (Fig. 2d–e). Marine resources probably always had some role in the diet of semi-enclosed and island regions such as Denmark and wider Southern Scandinavia, but were of much reduced importance after the introduction of agriculture (Figs. 2i and 4); fish bones, for example, are much rarer in Neolithic deposits than Mesolithic^12^. There is only sporadic appearance of shell middens after ca. 4200 BP, and they disappear completely from the landscape after ca. 3700 BP at the beginning of the Bronze Age^13^, as agriculture intensified and marine productivity declined (Fig. 2d). Shell middens remain absent until the Roman Warm Period. These early Iron Age (ca. 2500–1600 BP) middens are dominated by blue mussels^13^ (*Mytilus edulis*) and associated with a very different set of socio-economic conditions and population dynamics.

## Discussion

We show that the HTM in northern Europe and a simultaneously higher sea level fuelled increased coastal marine production in two periods, P1 (ca. 7600–7100 BP) and P2 (ca. 6400–5900 BP), which we argue resulted in abundant marine resources and enabled regional population increases across two distinct phases prior to the introduction of agriculture (Fig. 4). The mechanisms behind increasing productivity are likely complex, but include nutrient input from the catchment during sea-level transgressive phases immersing the land^33,35^, increasing temperatures during the HTM^36,37^, increased input of nutrient-rich oceanic water from the North Sea under higher sea levels and internal loading of nutrients following turnover in deep stratified systems (e.g. Lewis et al.^39^). Due to an increased area of connection with the North Sea under higher sea levels, the Kattegat, Limfjord and Danish coastal waters receive greater input of (nutrient rich) highly oxygenated marine water from the Skagerrak/North Sea, increasing both salinity and productivity within these waters. Higher palaeoproductivity has also been documented in Skagerrak sediments during warmer periods such as the Medieval Climate Anomaly and associated with the positive state of the North Atlantic Oscillation (NAO)^50^. The long-term history of the NAO remains equivocal, though conditions consistent with a predominately positive NAO state, particularly increased storminess, higher sea levels and higher temperatures across northwest Europe^51^, have been inferred over the HTM^52,53^, and could also be responsible for driving nutrient-rich water into Southern Scandinavia and/or increased marine production.

Prior to the introduction of agriculture intense marine resource exploitation, persisted for almost two millennia (ca. 7600–5900 BP), though particularly focussed between ca. 7600–7100 BP (P1) and ca. 6400–5900 BP (P2; Fig. 2). This is shown here by the widespread development of large, accessible shell beds in Danish inner waters (Fig. 2e) and, by implication, other marine resources such as mammals, fish and birds^11,12,44^. We argue that our data support the contention that pre-agricultural hunter-gatherers actively chose to exploit a rich, easily accessible coastal environment rather than, at this time, less cost-effective terrestrial resources. It is plausible that the Kongemose and Ertebølle cultures whose diet was predominately marine-based^10^ were forced into exploitation of marine resources (at least to some degree) by reduction in terrestrial resources^38,45^ at least on the Danish islands^45^. The preceding Maglemose culture (ca. 11600–8400 BP) inhabited Southern Scandinavia during the dominant birch-pine-hazel forest^38^, and were likely endowed with a large fruit and nut harvest, in addition to a large population of terrestrial animals due to this terrestrial food surplus. Population dynamics over the Maglemose and early Kongemose period are poorly understood, but one can assume at least a gradual population increase (as seen in other nearby areas during the Early Holocene^23,25^), although based on the likely rich terrestrial food supply present during the Maglemose period, population increase might have been relatively rapid.

The transition to the primeval (lime-elm-oak) forest resulted in a reduction in the fruit and nut supply and poorer quality hunting grounds, culminating in the extinction of two key prey species, elk and aurochs, from some areas^45^. This might have resulted in a population packing problem, leading to the disappearance of the Maglemose culture and subsequent emergence of the Kongemose culture, who solved the terrestrial-protein stress challenge by utilising marine resources. Rapid sea-level rise associated with the Littorina transgression (ca. 9000–7600 BP) reconfigured the landscape, flooding large land areas and presenting plentiful marine resources easily accessible to these coastal dwelling communities. This sea-level rise, which reached its maximum around ca. 7600 BP^34^, changed the Southern Scandinavian area from a large contiguous land area, land-settled with England and southern Sweden, largely to a group of islands, which must have had a huge impact on the resources available, in addition to vegetation change. For example, increased pressure on elk and auroch populations confined to the Danish islands following sea-level rise and subsequent land transformation likely led to their extinction from the Danish islands, whereas these species did not disappear from Jutland, which remained connected to the continent^45^.

Terrestrial protein was available to the later Ertebølle culture (ca. 7400–5900 BP) during the productive HTM, as shown by the presence of a diverse range of terrestrial animal remains at Ertebølle archaeological sites^11^, but it is plausible that a bountiful marine environment was actually a more cost-effective strategy due to dense forest cover and coastal topographical conditions which also allowed easy access from the sea or along the coast. The natural environment of Southern Scandinavia is very different to most habitats where marine protein dominates (e.g. cold regions in high latitudes^27^), with micro-tidal, low-energy shallow conditions making exploitation of the marine environment far easier. This was likely an initial draw to this environment, with technology such as boats and nets coming later as these peoples really mastered their environment. The lack of relief also means that river systems are generally small, slow flowing and easily accessible, meaning that freshwater and marine resources can be gathered in close proximity, with available plant resources and terrestrial animals utilised as supplements. The openness of the Mesolithic forest is hotly debated^54^, but as indicated above, under the closed canopy theory^55^ poorer terrestrial hunting grounds and highly seasonal/widely spaced plant resources might have lowered the cost-effectiveness of terrestrial resources, at a time when marine resources were abundant and easily accessible.

The onset of agriculture widened the economic resource base, sustaining a greater population density, through progressively landscape-scale exploitation of terrestrial resources (Figs. 2g–i and 4). This, in turn, had profound impacts on the human-environment relationship, with technology and culture increasingly buffering direct environmental effects (Fig. 4). However, climatically-driven changes in the coastal environment prior to this (ca. 7600–6000 BP) provided opportunities for cultural development/expansion including maritime technological adaptation and population growth, particularly focussed over the two high marine production events (Fig. 2). At these times hunter-gatherer populations made extensive use of prolific coastal resources across Southern Scandinavia, and between ca. 6600 and 5900 (spanning P2, ca. 6400–5900 BP; Fig. 2) relied on this mode of subsistence over agricultural practices up until the eventual disappearance of the Ertebølle people and migration of the first farmers to Southern Scandinavia^46^. Successful exploitation of available natural resources by hunter-gatherers can delay or prevent the need for agricultural innovation, while threshold changes in abundance or accessibility (due to environmental change or overexploitation for example) can set the stage for rapid cultural change involving an alternative strategy (such as the widespread and largely synchronous Neolithisation of Scandinavia around 5900 BP).

Whilst the agricultural revolution is clearly important to later population increase in Southern Scandinavia and elsewhere, this study further highlights that under the right circumstances, populations can grow and societies develop on predominately marine resources. It further highlights the importance of new evidence concerning the ongoing debate as to whether the Southern Scandinavian Ertebølle culture can be considered a complex society^56^. Societal complexity is a broad topic beyond the scope of the data presented here, but some of the common characteristics used to measure complexity are met by the Ertebølle culture, including expanding populations, technological development to enhance exploitation of the environment^44^ (Fig. 2g), and possibly some form of societal structure (as suggested by numerous ritualistic Mesolithic burials including animals and ornaments^57^). Furthermore, whether the Ertebølle culture were sedentary (or at least quasi-sedentary) remains debated^56,58^. Based on these combined factors, the Southern Scandinavian Ertebølle culture has previously been compared with the complex foragers of northwest America^58,59^ and may further challenge the axiom that agriculture is necessary for the rise of complex societies^60^.

The data presented here further highlight the global significance of the debate concerning the long-term growth and development of specialist coastal communities based on freshwater/maritime resources, rather than classically more cost-effective terrestrial resources. The continued discovery and excavation of coastal archaeological sites, production of meta-datasets^7^ (e.g. Fig. 2e and f) and comparison with focused multidisciplinary (and multiproxy) data detailing environmental and climate change will help to reveal multifaceted environmental-cultural interactions, maritime adaptations and perhaps in some cases further demonstrate multiple pathways to societal complexity. Such multidisciplinary studies will also begin to address a number of key questions that classic archaeological investigation has only been able to infer speculatively. In a broader sense, this study contributes to several of the “grand archaeological challenges” outlined by Kintigh et al.^6^ (particularly challenges A5, E2, E5 and E7), concerning the development of small-scale communities into larger, more complex societies (A5), the drivers behind population growth (E2), delayed emergence of agriculture (E5) and long-term human-environment interactions (E7). This study also contributes to our understanding of key issues in global environmental change, by demonstrating important linkages between sea level, climate (particularly temperature), marine production and resource availability in the past, that are of relevance for the present day and future. The HTM may provide a key analogue for changes in marine productivity expected under future global warming and rising sea levels (increasing exchange of oceanic water), exacerbated by major terrestrial exports of nutrients to coasts world-wide^61,62^ with implications for coastal resource availability for modern societies.

## Methods

### Coring and sediment core chronology

Following core collection, lithological description, physical analyses (i.e. loss-on-ignition) and subsampling of sediment cores for proxy analyses were undertaken for each sequence (see Supplementary Information Note 1 and Supplementary Table 1) except Korup Sø (KS). From each sediment core, plant macrofossils were picked from wet sieved fractions (500 and 100 µm) taken from core slices (1–2 cm thick over the study period, for all sites except Norsminde Fjord (NF) 8–10 cm slices) and KS (no plant macrofossil analyses performed). Plant macrofossils and molluscs were picked and identified from both fractions (see below) and the finer fraction (i.e. 100 µm) was retained for foraminiferal analyses (all sites except KS as no material from this core sequence now remains). Age-depth models (see Supplementary Note 1 and Supplementary Tables 2 and 3) were produced using ^14^C dates from terrestrial plant macrofossils (or molluscs for KS only).

### Coastal environmental proxies

Sedimentary pigment samples for Kilen (Kil) follow methodologies described in Lewis et al.^39^. At Horsens Fjord (HF) and Tempelkrog (TK), sedimentary pigment preparation and analyses follow methods outlined in Leavitt and Hodgson^63^. For all sites, diatom, foraminifera and mollusc sample preparation and analysis follow techniques described in Lewis et al.^39,40^. Diatom-inferred salinity was quantitatively inferred using a WA-PLS-component 2 model (*r*^2^ = 0.87, RMSEP = 0.44 square root units; bootstrapping x1000 cycles), based upon a trans-Baltic modern training set; see Lewis et al.^39,40^. Further details are provided in Supplementary Information Note 1 and original proxy datasets are provided in Supplementary Figs. 1–18.

### δ^13^C, δ^15^N and δ^2^H isotope analyses

Stable isotope values were obtained from bone collagen from human remains and preparation/analyses follow procedures outlined in van der Sluis et al.^47^ (see Supplementary Note 1 and Supplementary Dataset 1).

### Human resource exploitation and shell midden abundance

To represent relative human marine resource exploitation over the study period we use shell midden abundance, presented here using the summed probability distribution (SPD) of 231 calibrated ^14^C dates on *Ostrea edulis* shells present in Danish shell middens (Fig. 2e). To test the significance of the SPD, we calculated an average SPD from 1000 simulated datasets. Each dataset contained 231 samples with an error distribution similar to the real ^14^C data which were randomized using a uniform probability distribution in calendar years ranging from 8100 to 3500 BP (i.e. beyond the range of the study period, 8000–4000 BP). Further details are provided in Supplementary Note 2.

## Supplementary information

Supplementary Information

Description of Additional Supplementary Information

Supplementary Dataset 1

## Data Availability

All data are available in the main text or the Supplementary Information and can be supplied by contacting the corresponding author (Jonathan Lewis).
