# Supplementary material for: Marine resource abundance drove pre-agricultural population increase in Stone Age Scandinavia
Source: Nat Commun. 2020 Apr 24;11:2006. doi: 10.1038/s41467-020-15621-1 (PMC7181652; doi:10.1038/s41467-020-15621-1)
Supplement: Supplementary file 1 — Supplementary Information [file 41467_2020_15621_MOESM1_ESM.pdf]

Supplementary Information for

Marine resource abundance drove pre-agricultural population increase in Stone Age Scandinavia

Lewis, Ryves et al.

Supplementary Notes 1

Study sites, background information and methods for palaeoenvironmental reconstruction from Danish coastal sediments

Danish coastal palaeoenvironmental study sites

Data from six coastal sites (Kilen, Sebbersund, Korup Sø, Norsminde Fjord, Horsens Fjord and Tempelkrog) are presented in the main text (see also Supplementary Figures 1-19 and Supplementary Figure 21). Further details about each site are provided in Supplementary Table 1. In Supplementary Figure 1, sediment accumulation rate data are provided for all sites. An additional off-shore site (in Aarhus Bay) is also included as this sediment core was collected almost adjacent to Norsminde Fjord and demonstrates efficient oceanic exchange of sediment (and by inference other materials) during the late Mesolithic period (ca. 8000-6200 cal. yrs BP; hereafter BP unless otherwise specified); i.e. sediment is being removed from the coastal waters and transported into the deeper basins offshore. The Aarhus Bay sediment accumulation data form part of an independent marine environmental change project[1] and is presented as supplementary information with permission of the authors.

Danish coastal analyses: chronology, additional methodology and proxy records

Accelerator mass spectrometry ^{14}C dating and age-depth models

Prior to dating and age-depth modelling, core sequences were correlated via physical analyses (organic, carbonate and minerogenic matter; Supplementary Table 1), using loss-on-ignition techniques outlined in Dean et al. and Hieri et al.[2, 3]. From the finalised core sequences, selected samples were sieved and picked for plant macrofossils for Accelerator Mass Spectrometry (AMS) ^{14}C dating. Appropriate material selected for AMS ^{14}C dating were analysed at the Aarhus AMS Centre (AARAMS) at Aarhus University (using an EN tandem accelerator) or at the 14CHRONO Centre, Queen's University Belfast (UBA-numbers in supplementary data). ^{14}C dating procedure follows methods outlined in Lewis, Philippsen et al. and Olsen et al.[4, 5, 6]. Results were reported as conventional radiocarbon dates (^{14}C yrs BP, before 1950 AD[7]) and calibrated into calendar years using the IntCal13 calibration curve[8]. At Korup Sø no plant macrofossil analyses were performed[4, 9] and therefore the age-depth model for this site is based on 7 mollusc samples (5 dated at the Copenhagen ^{14}C laboratory and 3 at the AMS ^{14}C Dating Centre at Aarhus University[4, 9]; with a 400±50 year marine reservoir correction applied to each shell sample and calibrated with Marine13[8]. The *Ulmus* decline evident in the pollen record was used to verify the

radiocarbon chronologies at Kilen, Norsminde Fjord and Korup Sø (with pollen samples being prepared using standard techniques outlined in Fægri and Iversen[10]). No pollen data are available for Sebbersund, Horsens Fjord and Tempelkrog. Subsequent age-depth modelling was performed in either OxCal[11, 12] or Bpeat [13] (see Supplementary Table 2 and below for further details).

A brief summary of the chronological records is provided in Supplementary Table 2. Age-depth models for all sites except Sebbersund are available in Lewis et al.[14] and supplementary information. The AMS ^{14}C analyses for Sebbersund are provided in Supplementary Table 3 and the age-to-depth model was generated using OxCal 4.3[11] (Supplementary Figure 19).

Over the study period (8000-4000 BP) each site exhibits a continuous sequence with no evidence of hiati, though the Korup Sø record finishes at ca. 4200 BP, due to sediment infilling and isostatic uplift processes[4, 9]. Lithology for all sites is largely homogenous (marine silty-clay gyttja, with low organic content) between ca. 8000-3700 BP. Plant macrofossil and mollusc remains (Supplementary Figures 11–15) are present in all sequences though abundances vary markedly both within, and between sequences. Bulk sediment accumulation rates (AR) (Supplementary Fig. 1) were calculated for each site based on the AMS ^{14}C age-depth models for all sites. These age-depth models allowed for changes in the AR at times of marked change in physical sediment parameters (organic matter, $CaCO_3$ and minerogenic -AR) to provide a more realistic picture of sedimentation and avoid over-reliance on the ^{14}C dates as indicators of sediment rate change[6]. See references in Supplementary Table 1 for more detailed descriptions of the lithology, sedimentation rates and physical parameters. For Sebbersund, further details are available on request.

Diatoms and diatom salinity model

The diatom assemblages, diatom-inferred salinity (DI-salinity; in g L^{-1}), salinity deviation from modern and diatom flux data for each site are shown in Supplementary Figures 5-10. Diatoms samples for each site were prepared using standard digestion, washing and (Naphrax) slide mounting techniques[15, 16, 17], with microspheres added for diatom concentrations[18]. A minimum of 300 valves were counted per sample, excluding *Chaetoceros* resting spores and *Skeletonema* valves. The DI-salinity model incorporates 210 surface sediment training sites (from the Baltic Sea and Kattegat, predominately from Denmark, Sweden and Finland), incorporating 309 species and spanning a salinity gradient 0.5–31 g L^{-1} (A.L. Clarke, *et al.* unpublished data[4, 14, 19]). *Skeletonema* valves and *Chaetoceros* cysts were excluded from the diatom-salinity model as *Skeletonema* does not

usually preserve well and *Chaetoceros* spores cannot be reliably identified to species level. Salinity, depth, total nitrogen (TN) and total phosphorus (TP) were the only statistically significant variables (out of 11) explaining variation in the modern diatom dataset, explaining a combined 16% of the total variation. Salinity is the single most important variable (independently explaining 8% of the diatom data, with 9999 Monte Carlo permutations), with depth uniquely explaining 3.26%, and TN and TP accounting for 1.96 and 1.85%, respectively. Salinity shares almost no covariance with the other variables. Salinity was quantitatively inferred for each site using a WA-PLS-component 2 model (r^2=0.87, RMSEP=0.44, bootstrapping x1000 cycles, p<0.1) following testing for spatial autocorrelation within the training set[20, 21] via *h*-block cross validation using methods outlined in Trachsel and Telford[22]. DI-salinity values were converted to ‘deviations from modern salinity’ (for Fig. 2B; main text) based on modern average surface salinity values for each fjord (Supplementary Table 1) except Korup Sø and Kilen, due to both sites being isolated from the sea at present[4].

δ^{13}C, δ^{15}N and δ^{2}H isotope analyses

The bone collagen extraction protocol from Brock et al.[23], which is based on the Longin method[24] and revised with the inclusion of an ultrafiltration step[25, 26], was applied. Bone samples were cleaned and crushed into smaller pieces, followed by a demineralisation step (2% (0.6M) hydrochloric acid). The samples were dissolved at ~70˚C for 24 hours, followed by two filtering steps and lyophilisation. δ^{13}C and δ^{15}N values were measured using the Thermo Delta V IRMS with Flash 1112 Elemental Analyzer at the ^{14}CHRONO Centre in Queen’s University Belfast. δ^{2}H measurements were executed using the TC/EA coupled to a Thermo Delta V IRMS at the Stable Isotope Facility in the School of Planning, Architecture and Civil Engineering of Queen’s University Belfast.

Supplementary Notes 2

Archaeological and human-impact data

Shell midden abundance

Human marine resource exploitation over the study period is estimated via relative shell midden abundance (i.e. greater marine resource exploitation = more abundant are larger shell middens present). Our estimate is based on the summed probability of 231 calibrated ^{14}C dates on *Ostrea edulis* shells present in Danish shell middens (Fig. 2E; main text). There is substantial debate about the validity and methodology of using ^{14}C dates as a proxy for prehistoric activity[27, 28, 29, 30, 31, 32], but this method is being becoming more common[31, 32, 33, 34, 35, 36], and ultimately the reliability of this technique is determined by the quality of the dataset used. The dataset employed here is extremely comprehensive, incorporating 231 dates from 42 shell middens. This is a semi-quantitative measure, which we argue, in the absence of any other reliable method of quantifying shell midden abundance or volume, is representative of the general shell midden trend (in terms of presence and relative abundance) over the study period, agreeing with the widely accepted view among Danish archaeologists[37, 38]. We acknowledge that these data may be biased by potential focussing of scientific interest in the Mesolithic, abundance/availability of oysters to ^{14}C date and the absence of dates submitted to other dating facilities, but we believe that this is a valid proxy of midden abundance over this period, agreeing well with archaeological observation of trends over the study period[37, 38, 39]. Furthermore, the great majority of oyster shells submitted for dating were sent to either the Aarhus University or Copenhagen National Museum dating facilities.

Following their first appearance around ca. 7600 BP[40] in Denmark, shell middens gradually increase in size/volume and abundance, reaching their maximum extent between ca. 6400-5700 BP (based on the data presented here; Fig. 2e main text). They decline in abundance in the Early Neolithic, though remain continuously present up until ca. 4200 BP and sporadic until ca. 3700 BP, before disappearing completely in the Bronze Age. Danish shell middens from the late Stone Age can contain diverse mollusc assemblages, but classically contain abundant *Ostrea edulis* (European flat oyster), *Cerastoderma edule* (edible cockle), *Mytilus edulis* (blue mussel) and *Littorina littorea* (common periwinkle). Across the Mesolithic-Neolithic transition (ca. 5900 BP) many of these middens exhibit a distinct faunal shift in mollusc composition from oysters dominance in the Ertebølle layers (ca. 7400-5900 BP) to predominately cockles and mussels in the early Neolithic (Funnel Beaker layers), which we

have argued previously may be due to changing climate and sedimentary conditions[14]. Another pulse of oyster dominated shell middens coincides with the Pitted Ware-Single Grave Cultural period ca. 4400-4800 BP.

Shellfish exploitation is indicative of a wider phenomenon of marine resource utilisation that is visible in archaeological remains (particularly shell middens but also in other types of coastal sites/settlements). From the many comprehensively excavated middens across coastal Denmark, it is clear that in the late Mesolithic, humans exploited a wide range of marine resources in addition to shellfish, including marine mammals, birds and fish species (likely in addition to marine plants which have not been preserved). For example, in the late Mesolithic layers of the Bjørnsholm Bay shell midden, ~14 species of bird (marine and terrestrial/freshwater), multiple species of amphibians, >20 species of mammals (both terrestrial and marine) and >30 species of marine and freshwater fish were present in addition to a rich shellfish fauna[41, 42, 43].

From almost 9,000 fish bones from the Norsminde Fjord shell midden, over 25 different species were present[44], almost all of which were brackish or marine taxa (flounder most abundant, 57%). Flounder and gadids (e.g. cod and saith) are generally most abundant in settlements from the Vedbæk area, whilst in one of the northern most Ertebølle settlements (Yderhede) flatfish and spurdog bones dominate the assemblage[45]. Other sites, however, have demonstrated that fishing was not purely a marine activity, with fish species present from freshwater and fluvial sources. At Bjørnsholm Bay, catadromous eel bones were most abundant (56%), with other saltwater taxa accounting for 22% of fish bones[43].

The variability in fish bone composition between sites is somewhat contrasting to the relatively consistent shellfish composition of Ertebølle sites. Whilst fishing was clearly an important activity, particularly in the summer months[43, 45, 46], it appears that the catch (i.e. species exploited) was more strongly governed by localised factors, such as the topography, proximity to freshwater inlets or sites (e.g. rivers or lakes), water depth, protection, and availability of suitable breeding grounds (e.g. eel-grass beds, macrophytes). For example, it has been suggested that eel might have been readily caught at Bjørnsholm Bay, at the mouths of small streams running into the former fjord inlet, whilst at Norsminde, the shallow waters of the former Kysing Fjord might have provided ideal breeding grounds for flounder[43, 44, 45]. Whilst fish bones are often abundant in Mesolithic coastal shell middens and deposits, they appear to be very scarce in Neolithic layers (e.g. Bjørnsholm Bay, Mesolithic: Neolithic fish bone ratio, 11,490: 252 bones[43, 45], Norsminde Fjord, all but one bone were found in the Mesolithic layers[44]). The reason for this remains uncertain,

particularly as species composition and relative proportions (e.g. Bjørnsholm Bay), do not appear to change markedly from the Mesolithic layers[38, 45, 47, 48], suggesting similar fishing patterns (i.e. methods and targeted species). Site abandonment is ruled out due the clear continuation of numerous middens (and other sites bearing coastal resources) in the Neolithic (including some sites, dated entirely to the Neolithic period). It remains possible that fish bones were deposited elsewhere, but this is hard to believe considering the fact that both molluscs and bones from other animals were deposited on these sites. Perhaps accelerated decomposition of fish bones also occurred in the Neolithic layers, due to a reduction in shell midden accumulation rates (as marine resources became less important) and/or changes in the physical environment (e.g. increased aerial exposure, weathering and erosion). The most plausible explanation on a regional scale, however, is that the fish catch was substantially reduced due to introduction of agriculture and reduced dependence on (declining) marine resources[49, 50].

Isotopes and bones

Following the pioneering work of Tauber et al.[49], much research has been carried out into dietary trends of late Mesolithic/early Neolithic people from Denmark and south Scandinavia over the Mesolithic-Neolithic transition. Isotopic analyses (classically of $\delta^{13}C$ and $\delta^{15}N$) of bones and teeth have enabled reconstruction of the relative proportions of marine and terrestrially-derived dietary protein consumed by humans (and other animals) from coastal and inland sites across Denmark over the Holocene[49, 50, 51], demonstrating that humans consumed a predominately marine diet over the Late Mesolithic period (ca. 8,400-5900 BP), shifting abruptly to a more terrestrial diet at the onset of the Neolithic period (at ca. 5900 BP). The new data from the Limfjord presented in Fig. 2I (main text) adds further support to this trend, whilst also adding novel δ^2H data, providing new information on the marine component of diet[52]. The $\delta^{13}C$, $\delta^{15}N$ and δ^2H data are provided in Supplementary Dataset 1.

Population rise

Shennan et al.[33] (see also Downey et al.[32]) present relative population for northern Europe, broken down into 12 sub-regions, based on summed-calibrated radiocarbon date distributions across central and northwest Europe. Here, we focus on three of these regions (Jutland, Danish Islands and Scania) as these are most relevant to the present study region (i.e. south Scandinavia) and where there is clear evidence for intensive marine resource exploitation prior to the introduction of agriculture and a parallel population increase. In total,

these three records are based on 1019 radiocarbon dates (Jutland N = 409; Danish Islands N = 329; Scania N = 281). Prior to the population 'boom' in the early agricultural period[33], all three records show rising populations from approximately 6400 BP (Fig. 2, main text), which we show occurs in synchrony with a large increase in marine production (P2 in Fig. 2 main text). A simple series of one-tailed t-tests between the SPD values of each of the three regions over Population event 1 (Pop. 1; 7600-7100 BP) and Population Event 2 (Pop. 2; 6400-5900 BP) and the preceding 500 years for each population event (testing for unequal variances) shows that the SPD is significantly different ($p<0.05$) comparing Pop. 1 and Pop. 2 to the 500 years preceding each pulse for each region (see Supplementary Table 4). We further tested that the pre-agricultural population increase was a real phenomenon using the calibrated ^{14}C sample medians from the EuroEvol database[53] (Supplementary Figure 20). All ^{14}C dates from the Danish region were extracted from the EuroEvol database and calibrated using OxCal[11, 12]. The median was then extracted and used for constructing a histogram (light blue bars in Supplementary Figure 20) and subsequently plotted against the Shennan et al. [33] data on a secondary y-axis for comparison. Indeed the median histogram and the Shennan et al.[33] population curve follows each other closely. Hence, we argue that the increasing population prior to the Neolithic onset is real.

Some of the regions investigated by Shennan et al.[33] experienced agricultural innovation somewhat earlier than south Scandinavia (i.e. France, central and southern Germany). However, for the regions experiencing a later appearance of agriculture, several exhibit similar population increases (or pulses) in the late Mesolithic (i.e. northern Germany several pulses after ca. 7000 BP, but particularly after 6500 BP; Scotland, and western Sussex ca. 6500 BP). This suggests that similar population increases may have occurred further afield, potentially fuelled by abundant marine resources, though for these areas archaeological and/or marine environmental records are less comprehensive than those for south Scandinavia. For example, in Scotland there is some evidence for substantial marine resource exploitation from late Mesolithic shell middens[54, 55, 56] and a shift from a marine to terrestrial dominated diet[57, 58] across the Mesolithic-Neolithic transition. However, the archaeological shell midden records from Scotland are more ambiguous than those of south Scandinavia and, to date, no good quality marine palaeoenvironmental records detailing changes in marine productivity (e.g. long-term sedimentary pigment analyses) are available for Scottish coastal areas. Environmental hypotheses have been put forward to explain this potential dietary shift and the reasons behind the introduction of agriculture in Scotland[59], but variable regional climate data (including evidence for drier, wetter conditions and no change)

across the transition means that inferences of late Mesolithic/Early Neolithic climate remains contested and contradictory[55, 59, 60, 61, 62].

Technological innovation

The number of (marine) fishing technologies is based on data presented in Fig. 26 of Andersen[63]. We argue here that the increasing number of fishing technologies represents adaptation and development to exploit more efficiently the rich marine environment during the late Mesolithic. With the apparent rejection of agriculture (farming had reached central to northern Germany by ca. 6600 BP[64]), humans developed and innovated specialist technologies for exploiting the plentiful resources (including multiple fish, shellfish, marine mammal and bird species), thereby widening the economic resource base and enabling population to increase, prior to the introduction of agriculture. Similarly, terrestrial technologies developed to exploit the land are evident in the Neolithic period (particularly the polished flint axe and the primitive plough known as the ard[65, 66, 67] as agricultural practices evolved and became more intensive (with this expanding resource base supporting later pulses of population increase). Further information is provided in Supplementary Table 5.

Human impact on the landscape

Human impact is also acknowledged as an additional forcing factor that might drive changes in sedimentology and in biological assemblages (i.e. proxy data), particularly after the introduction of agriculture (ca. 5900 BP for Denmark[68]). This has been tracked here via comparison of the comprehensive land use change and agricultural development records available for Denmark (particularly local/regional pollen, plant macrofossils and sedimentation records). For two of the sites presented in this study (Korup Sø and Norsminde Fjord), full pollen records are available (Harald Krog and Peter Rasmussen, unpublished data) for which selected curves demonstrating human impacts in these catchments are presented in Supplementary Figure 21. The *Ulmus* (elm) decline provides a reliable and independent dating marker for Norsminde Fjord, Korup Sø and Kilen for verification of age-depth models (though this has only been tentatively placed for Kilen based on 5 pollen samples with lowered pollen/spore counts). The *Ulmus* decline has been shown to be widely synchronous across south Scandinavia and precisely dated via wiggle-matching and dendrochronology[68], though the underlying causes are likely spatially variable and highly debated[69].

Pollen records from Denmark generally show negligible human impact prior to the introduction of agriculture, with human impact and subsequent significant inputs of terrestrially-sourced material into the marine environment occurring in the post-agricultural period[69, 70, 71] (and see Supplementary Figure 21). The introduction of agriculture is generally marked by a decline in *Ulmus* pollen attributed to pathogen-related causes and/or human-forestry clearances for agriculture[68, 69, 72, 73, 74] (with substantial geographical variation). Later unambiguous agricultural indicators appear in pollen records, including *Plantago lanceoloata*, *Rumex acetellosa* (both common on agricultural land) and cultivated crops such as *Triticum* and *Hordeum*[71, 75]. Detailed reviews of the vegetation history of Denmark can be found in Iversen[73] and Odgaard[76].

The sedimentary records of lakes and fjords often show increased sedimentation accumulation rates (and/or peaks) and increased proportion of minerogenic matter following land-use change events[69, 70, 71] (including deforestation). The record for Lake Gudme Sø (Fig. 2H; main text) is an excellent example of how an aquatic system responds to increased sediment loads following land-use change for agricultural purposes in the Neolithic period, though there are many other examples available across the study region (e.g. "*landnam*" events[72, 73, 76, 77]). For the fjords presented here, some large-scale increases in sedimentation rate (and minerogenic content of sediment) are apparent in the Neolithic period (post 5900 BP; see Supplementary Figure 21), again likely linked to localised human impact on the catchment[4]. It is important to note that changes in the sedimentary records are often geographically variable due to topographical variations, localised catchment activity and varying sensitivity and response time of lakes/fjords to catchment changes. The key point here, demonstrated by both pollen and sedimentation records, is that during the Mesolithic period, there is little, or no, detectable impact on the land by humans, and it is only, after the introduction of agriculture that humans begin to manipulate the landscape more and more intensively to suit their needs.

Supplementary Notes 3

Regional records of Environmental Change

Temperature

The Holocene thermal maximum (HTM) has been shown to be global in extent, though the precise dates are geographically variable[78, 79, 80]. For northern Europe, it is generally accepted that the HTM occurs between 8000-4000 BP, with peak temperatures between ca. 7500-6000 BP[81, 82, 83], associated with intensification of the thermohaline circulation, northward migration of the polar vortex and persistent development of summer anticyclonic conditions over Scandinavia[79, 80, 83, 84]. To show the HTM in the main text, pollen-inferred average air temperatures for Lake Trehörningen pollen record (from south Sweden) are plotted in Fig. 2A (main text). Lake Trehörningen exhibits rising temperatures in the period after 8000 BP, reaching maximum Holocene temperatures between ca. 7500-6000 BP, prior to a steady decline after ca. 6000 BP, falling more rapidly after ca. 5500 BP (Fig. 2A main text), particularly in winter air temperature[81]. This curve has been chosen as we believe it is representative of the wider south Scandinavia area, the site is situated in close proximity to the study area and is relatively unaffected by human activity even in the Neolithic section of the record (i.e. post 6000 BP).

Whilst we feel this is the most appropriate temperature record to display in Fig. 2A (main text), it is important to clarify that similar conditions over the HTM are apparent in a number of other pollen records from south Scandinavia[84, 85, 86, 87]. Of note here is the composite Holocene reconstruction curve for northern Europe from Seppä et al.[81] (see below) and a more recent reconstruction for Denmark by Brown et al.[87], though we decided against plotting the Brown et al.[87] temperature reconstruction because Denmark has been shown to be heavily human-impacted following the introduction of agriculture[69, 71, 72, 73]. The Seppä et al.[81] record shows consistently higher temperatures between 8000-4000 BP across northern Europe in quantitative reconstructions from 36 widespread stacked pollen records (supported by stacked chironomid temperature inferences), and therefore supports the Lake Trehörningen record, though is less specific to south Scandinavia than Lake Trehörningen[82].

Higher temperatures during the HTM are further supported by characteristic floral and faunal changes throughout the study period[88, 89] (Supplementary Figure 22), particularly by the widespread presence of a number of warmth-demanding plants and animals[46] some of which are no longer present (e.g. Lusitanian ostracod species (*Aurila arborescens* and *Callistocythere badia*[89]), the European pond turtle *Emys orbiculare*[90], the warm-water

anchovy *Engraulis encrasicolus*[46] and the Aesculapian snake, *Zamenis* (previously *Elaphe*) *longissima*[91] in southern Scandinavia. A comprehensive (though somewhat older) review of conditions during the HTM in northern Europe is provided by Snowball et al.[83], where a wide-range of supporting evidence for highest Holocene temperatures between ca. 7500-5500 BP including glacial limits and variations, tree lines, sea-surface temperatures, palynological (pollen, plant macrofossis and megafossils) and palaeolimnological evidence (e.g. lake levels, biological proxies) is dicussed.

A notable exception is a record from the SE Gotland basin which suggests a late HTM, with cold, unstable conditions between 7000-6000 BP and maxima occurring between 6000-4500 BP[92]. Cooler conditions prior to the introduction of agriculture (7000-6000 BP) is in contrast to the vast majority of temperature data available for Southern Scandinavia[81, 82, 83, 87] (and Supplementary Figure 22). We also highlight that this temperature data is from sediment core analyses from the remote Gotland Basin, and so less relevant to the shallow Danish and wider Southern Scandinavia coastal/marine waters. Warden et al.[92] infer that rising temperatures ca. 6000 BP drive agricultural innovation and subsequent population growth[92]. In support of their SST reconstruction, Warden et al.[92] draw attention to two records in particular which show minor secondary increases in temperatures between ca. 6500-5700 BP and peaking around 5800-5700 BP[93, 94]. However, in contrast to Warden et al.[92], generally high temperatures precede this Early Neolithic peak in temperature in these two records, unlike the cold, unstable conditions apparent between 7000-6000 BP in the TEX_{86} SST reconstruction from the SE Gotland basin. Furthermore, it is important to highlight both problems/variability associated with SST reconstructions from biomarker techniques such as TEX_{86}[95, 96] and that these supporting records[93, 94] are somewhat selectively chosen from a much larger body of evidence that shows the HTM peak temperature occurring between 7500-6000 BP[81]. Lastly, in these two records rising temperatures pre-date the introduction of agriculture and agree well with the early population increase hypothesized here and might even provide a supporting mechanism for increasing productivity beginning ca. 6400 BP. It is acknowledged here that in some regional temperature reconstructions, winter temperature do not peak until later, but in south Scandinavia, the presence of thermophilous species (e.g. ivy, holly and mistletoe) in the late Mesolithic period, suggest that winters must have been relatively mild[73, 85].

Sea-level change

The selected sea-level curve (in Fig. 2C of the main text) is from the Blekinge region of southern Sweden, chosen as it is broadly representative of the wider area and is one of the most comprehensive and well-dated records available for the mid-Holocene from southern Scandinavia. This curve is based on multiproxy evidence (magnetic parameters, sediment stratigraphy, organic carbon, pollen, plant macrofossils, diatoms) from two well dated cores, and from a region that has experienced similar rates of uplift (i.e. rebound) to northern Denmark and Zealand since the deglaciation of the Fennoscandian ice sheet[97, 98, 99, 100]. Comparison of the Blekinge sea-level curve with other regional sea-level data shows good overall agreement in terms of major trends, though subtle differences do occur due to regional differences in climate causing 'piling up' of seawater[101, 102] and rates of isostatic rebound[103, 104, 105, 106]. In general, sea levels were higher than present day (+4 to +8 m above present in the southern Baltic and Denmark[100, 107, 108]) throughout the study period (8000-4000 BP). The broad pattern is characterised by rapid rise between 8000-7000 BP (particularly around 7600 BP[109]) associated with the marine (*Littorina*) transgression and global sea-level rise[110], reaching maximum levels between 6700-6000 BP, followed by a gradual decline after 6000 BP. Super-imposed upon this general trend are transgressive and regressive events (~1-3 m magnitude[106, 107]), though these may vary spatially in number and/or amplitude (Fig. 2 main text) due to local rates of isostatic uplift[104, 111] and climate conditions[101].

Supplementary Figures

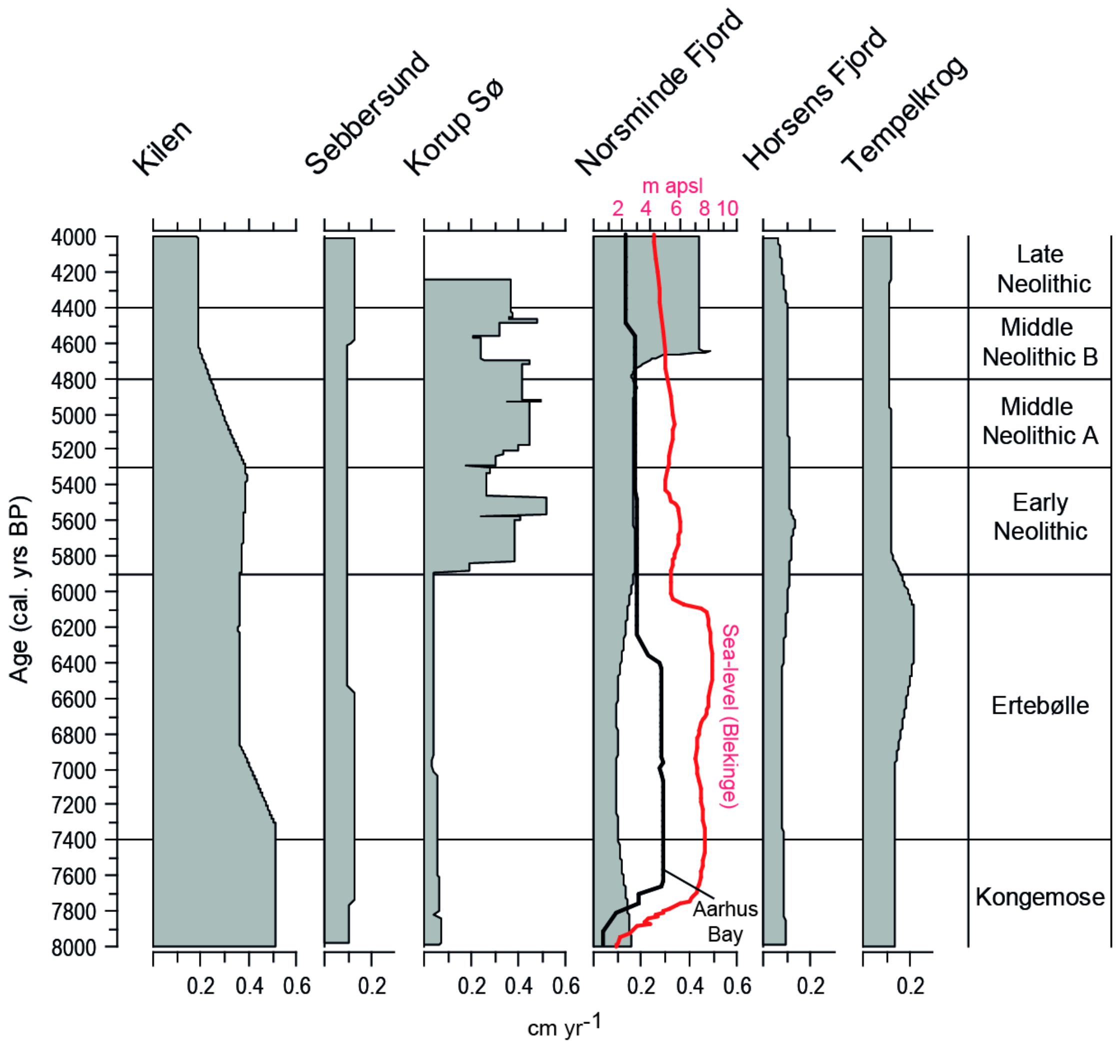

Supplementary Figure 1 I Sediment accumulation rates at all six Danish coastal study sites presented in this study. For Norsminde Fjord, the accumulation rate at an almost adjacent offshore site in Aarhus Bay[1] is also presented, along with regional sea level[107], to demonstrate the anti-phase relationship over the Mesolithic late Kongemose and Ertebølle periods (i.e. lower accumulation in the inner coastal waters and higher sedimentation in offshore waters). This is interpreted as increased efficiency of water and sediment exchange between the inner coastal water and the more open offshore areas between 8000-6200 BP under times of higher sea level. Sediment (and likely other material) is being transported from the coastal areas and deposited in the deeper sea basins, hence slower accumulation rates are often apparent in coastal sites prior to the introduction of agriculture (e.g. Norsminde Fjord, Korup Sø, Horsens Fjord) and high accumulation rates are evident offshore. This is important for some mollusc taxa (e.g. *Ostrea edulis*) that favour coarse silt/sand substrates, with little vegetation cover and can be choked under conditions of fast accumulation of fine sediments[112, 113].

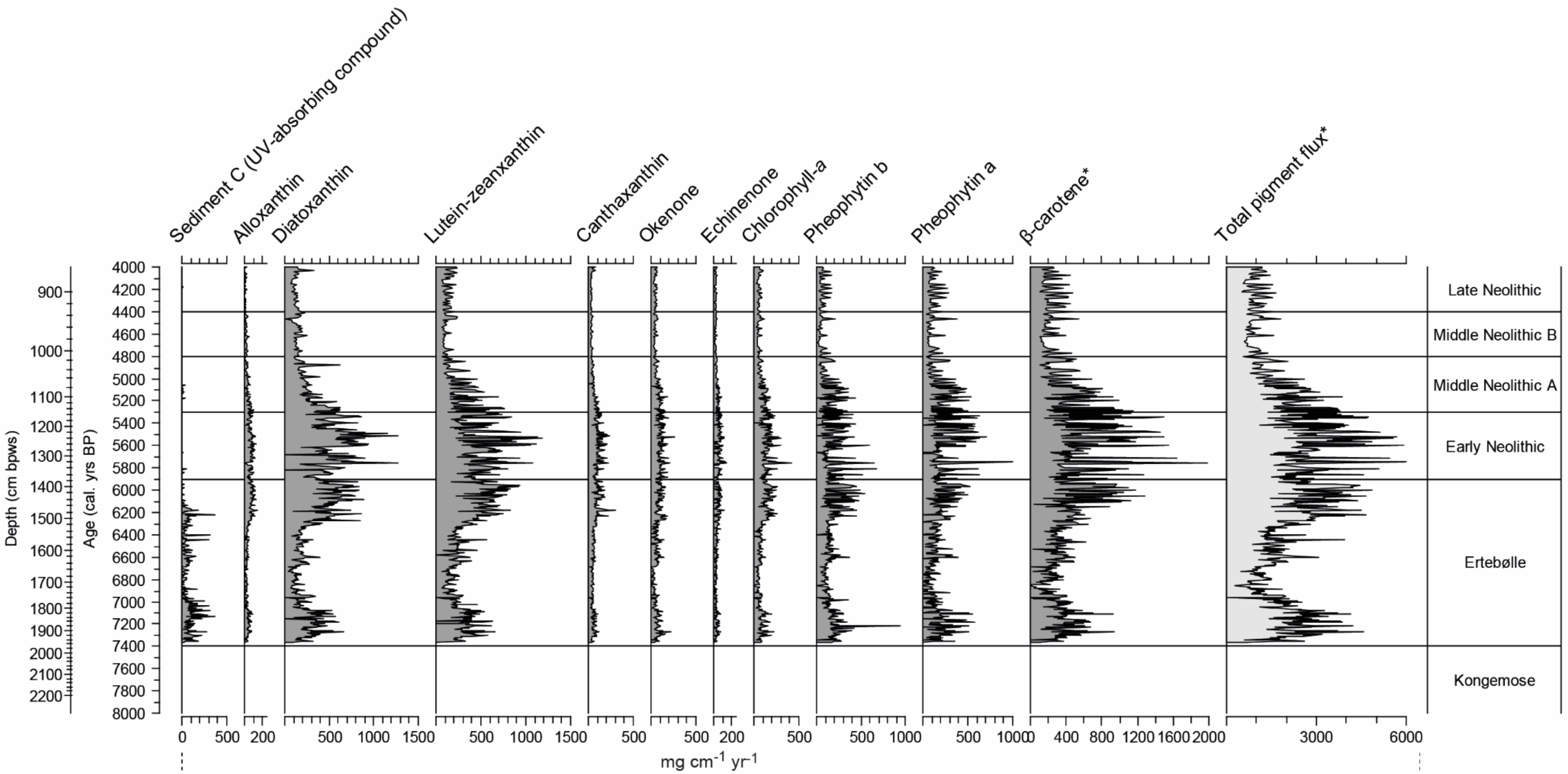

Supplementary Figure 2 I Kilen sediment pigment flux data. Pigments were identified based on their retention time and absorption spectra compared with published literature (e.g. Jeffrey et al.[114]) and authentic standards. *[asterix] denotes that the plot is displayed on independent scaling to other like plots to improve visual display. Sub-divisions follow archaeological periods in Fischer and Kristiansen[115]. Further discussion provided in Lewis, Lewis et al.[4, 19]. Analyst: Jonathan Lewis.

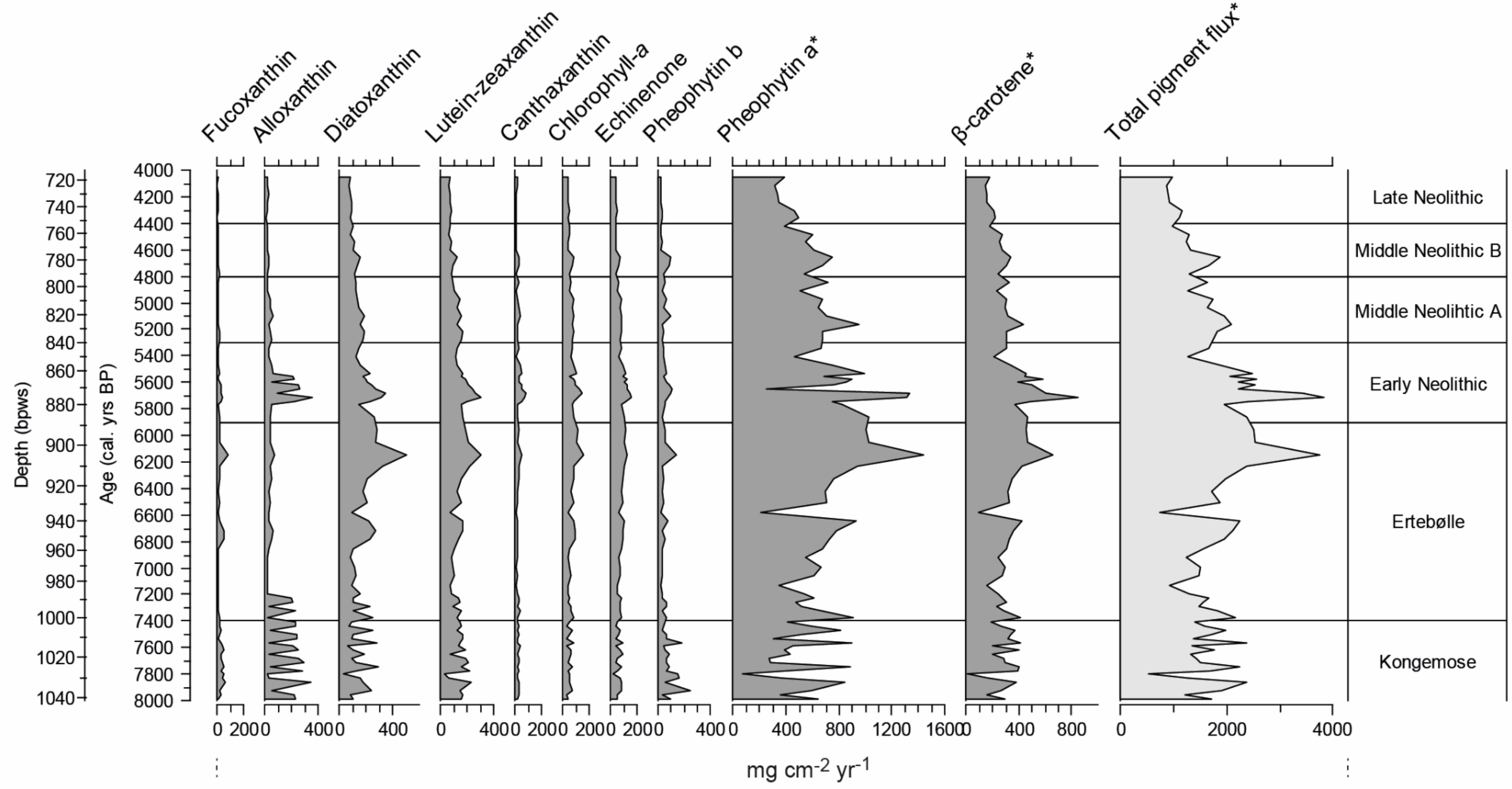

Supplementary Figure 3 I Horsens Fjord sediment pigment flux data. Pigments were identified based on their retention time and absorption spectra compared with published literature (e.g. Jeffrey et al.[114]) and authentic standards. *[asterix] denotes that the plot is displayed on independent scaling to other like plots to improve visual display. Sub-divisions follow archaeological periods in Fischer and Kristiansen[115]. Analyst: Rolf Vinebrooke.

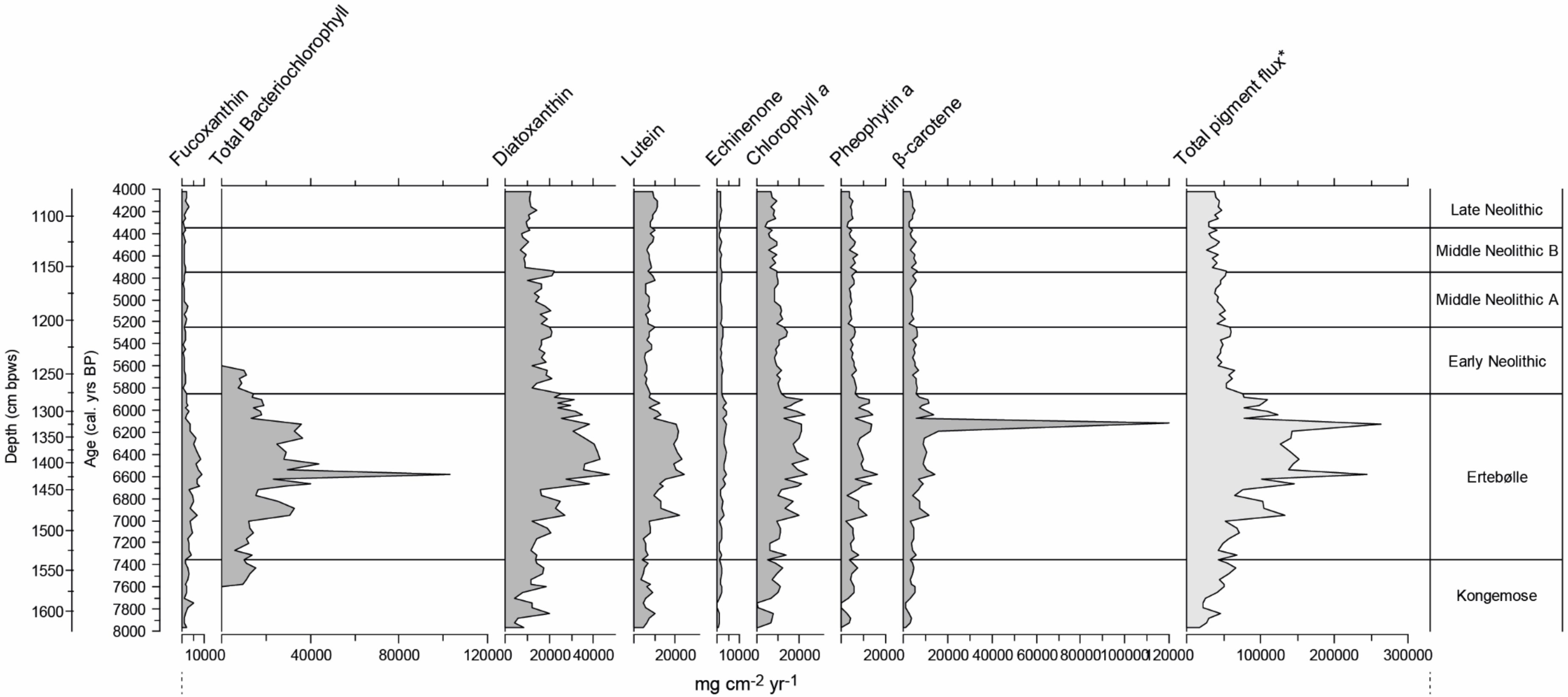

Supplementary Figure 4 I Tempelkrog sediment pigment flux data. Pigments were identified based on their retention time and absorption spectra compared with published literature (e.g. Jeffrey et al.[114]) and authentic standards. *[asterix] denotes that the plot is displayed on independent scaling to other like plots to improve visual display. Sub-divisions follow archaeological periods in Fischer and Kristiansen[115]. Analyst: Rolf Vinebrooke.

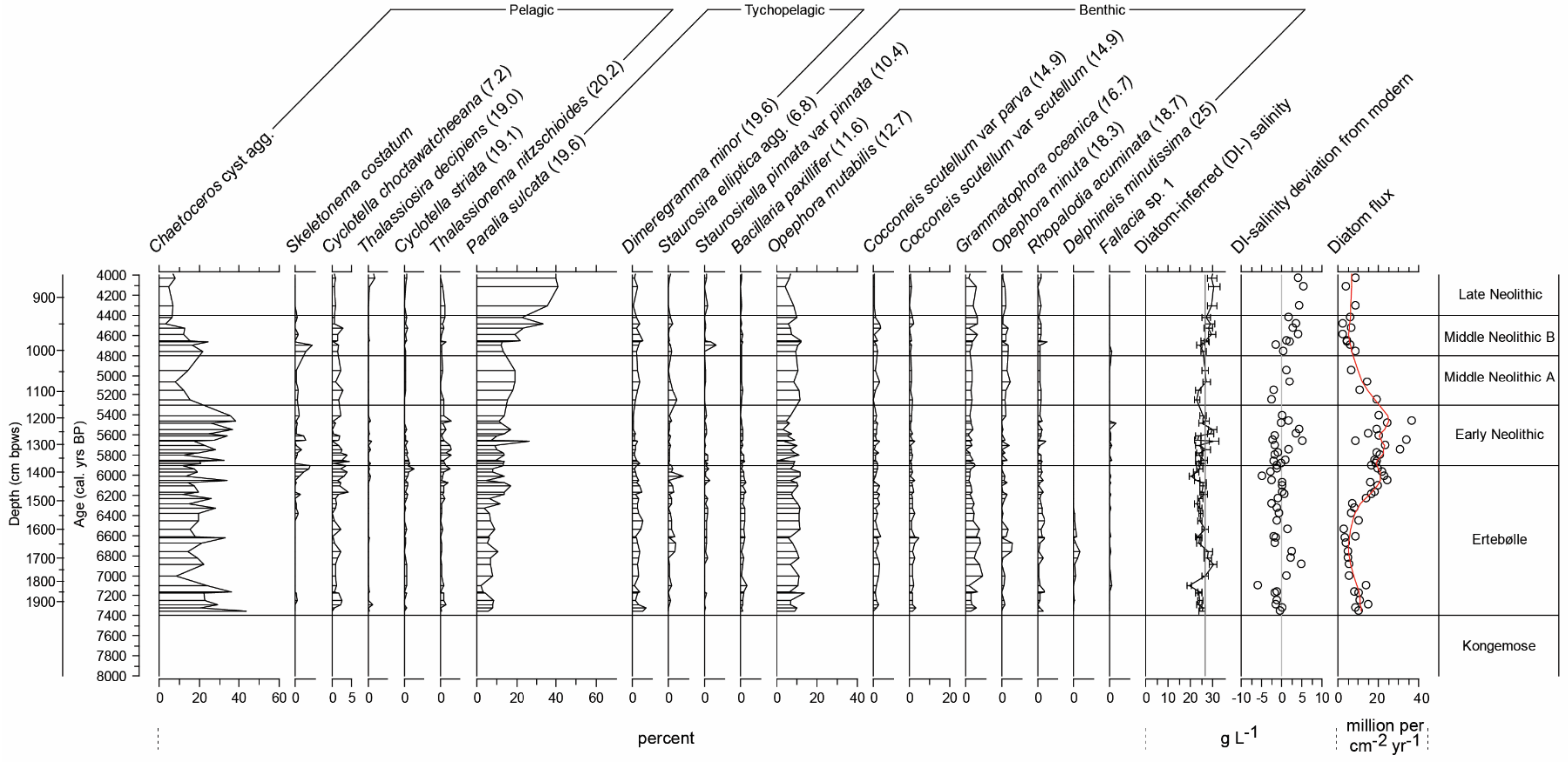

Supplementary Figure 5 I Kilen diatom assemblage (selected species > 3%). Associated metrics include diatom inferred (DI-)salinity (with sample-specific standard errors (via bootstrapping) based on a pan-Baltic 210 site training set; Supplementary Notes 1), DI-inferred salinity deviation from modern (taken from adjacent Struer Bay 25.5 g L^{-1}, based on Burman and Schmitz[116] and Hoffmeister et al.[117]) and diatom flux (with loess smoother; 0.1 span). Diatom data expressed as percentages of the whole assemblage. The diatom assemblage has been ordered via basic habitat type (i.e. pelagic, tychopelagic or benthic) and within these classes via weighted-averaging salinity optima (i.e. values in brackets in g L^{-1}) from fresh/brackish (on left) to marine (on right) generated from the modern diatom training set (Supplementary Notes 1). Sub-divisions follow archaeological periods in Fischer and Kristiansen[115]. Further discussion provided in Lewis; Lewis et al.[4, 19]. Analyst: Jonathan Lewis

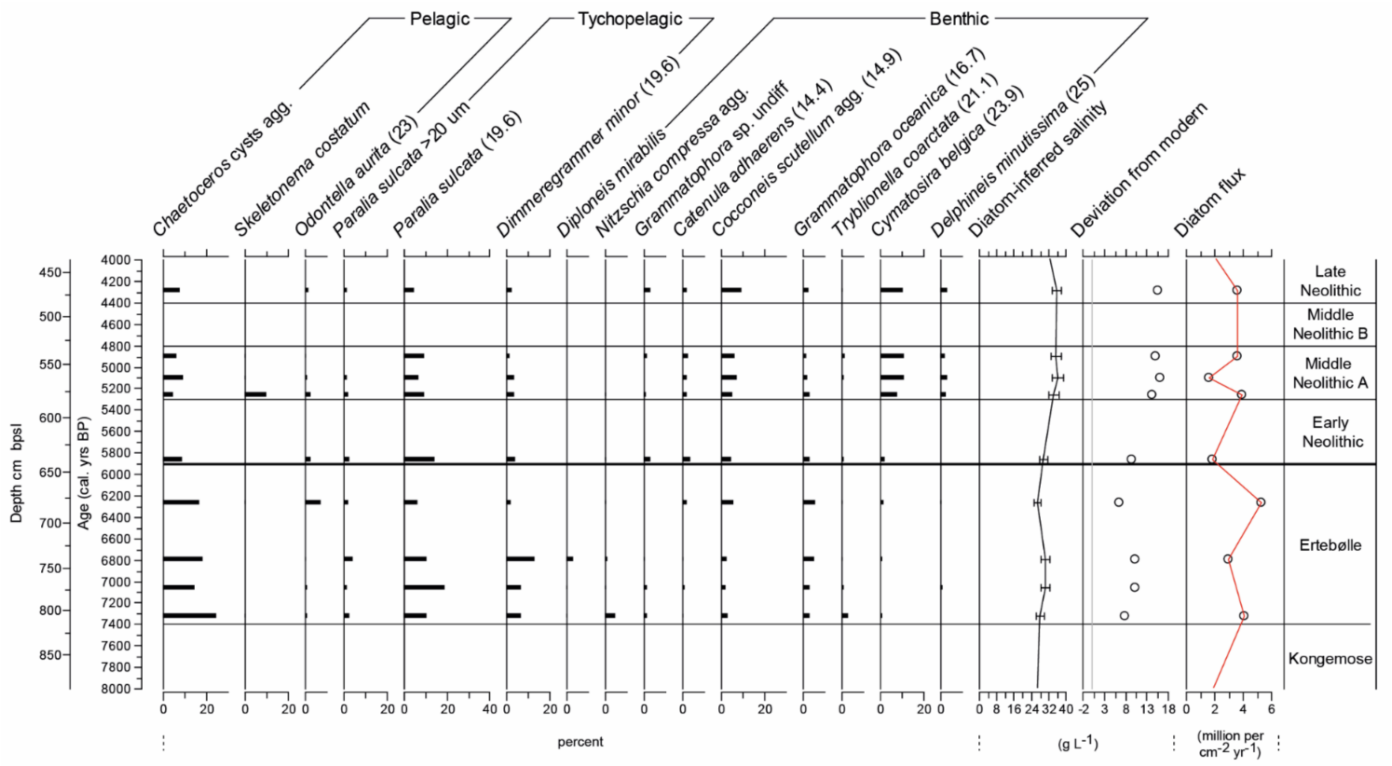

Supplementary Figure 6 I Sebbersund diatom assemblage (selected species > 3%). Associated metrics include diatom-inferred (DI-)salinity (with sample-specific standard errors (via bootstrapping) based on a pan-Baltic 210 site training set; Supplementary Notes 1), DI-inferred salinity deviation from modern (20.2 g L^{-1}[116]) and diatom flux (with loess 0.1 span smoother). Diatom data expressed as percentages of the whole assemblage. The diatom assemblage has been ordered via basic habitat type (i.e. pelagic, tychopelagic or benthic) and within these classes via weighted-averaging salinity optima (i.e. values in brackets in g L^{-1}) from fresh/brackish (on left) to marine (on right) generated from the modern diatom training set (Supplementary Notes 1). Sub-divisions follow archaeological periods in Fischer and Kristiansen[115]. Analyst: Jonathan Lewis.

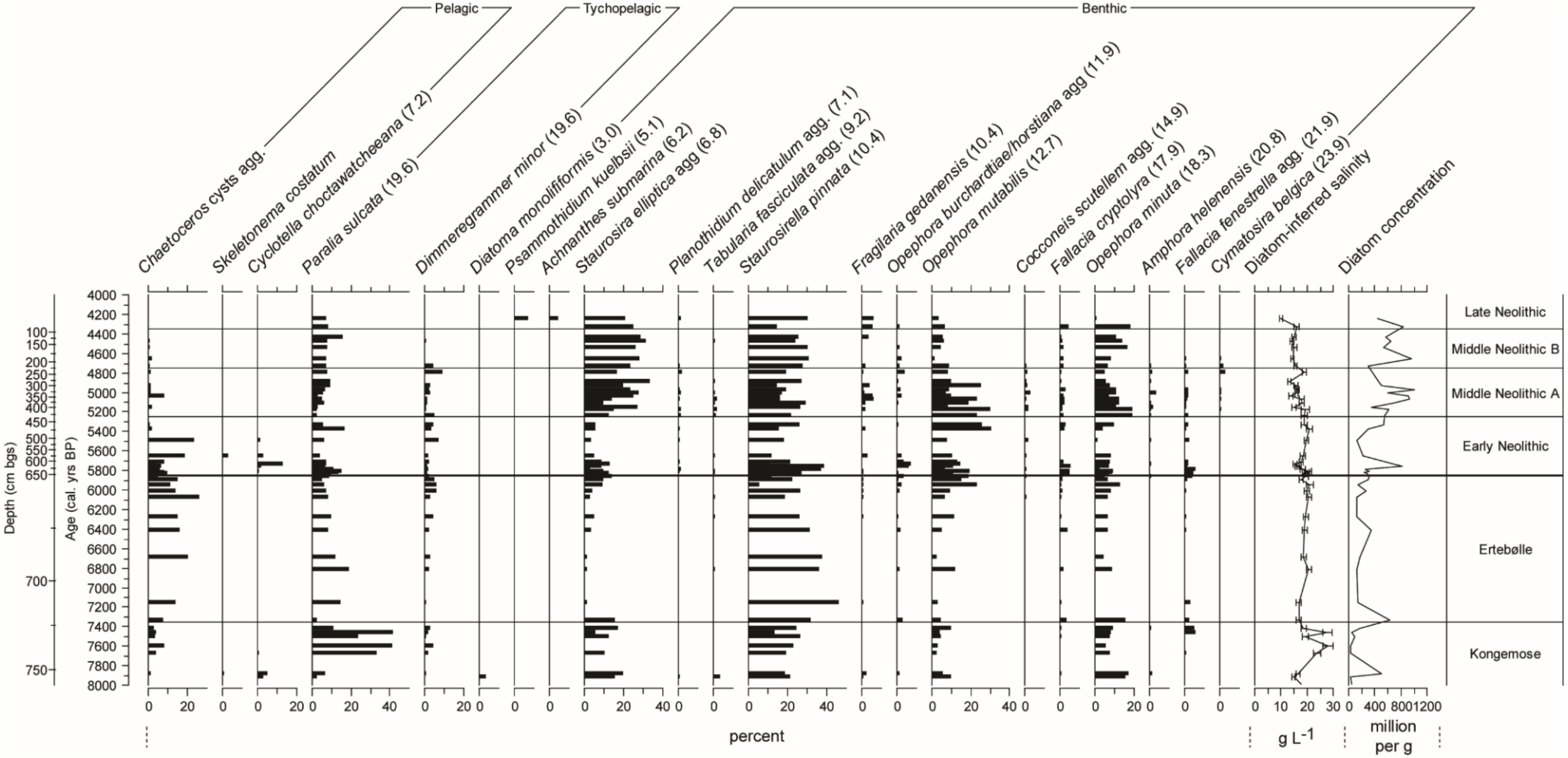

Supplementary Figure 7 I Korup Sø diatom assemblage (selected species > 3%). Associated metrics include diatom-inferred (DI-)salinity (with sample-specific standard errors (via bootstrapping) based on a pan-Baltic 210 site training set; Supplementary Notes 1) and diatom concentration. Diatom data expressed as percentages of the whole assemblage. The diatom assemblage has been ordered via basic habitat type (i.e. pelagic, tychopelagic or benthic) and within these classes via weighted-averaging salinity optima (i.e. values in brackets in g L^{-1}) from fresh/brackish (on left) to marine (on right) generated from the modern diatom training set (Supplementary Notes 1). Sub-divisions follow archaeological periods in Fischer and Kristiansen[115]. Further discussion provided in Lewis[4]. Analyst: Jonathan Lewis.

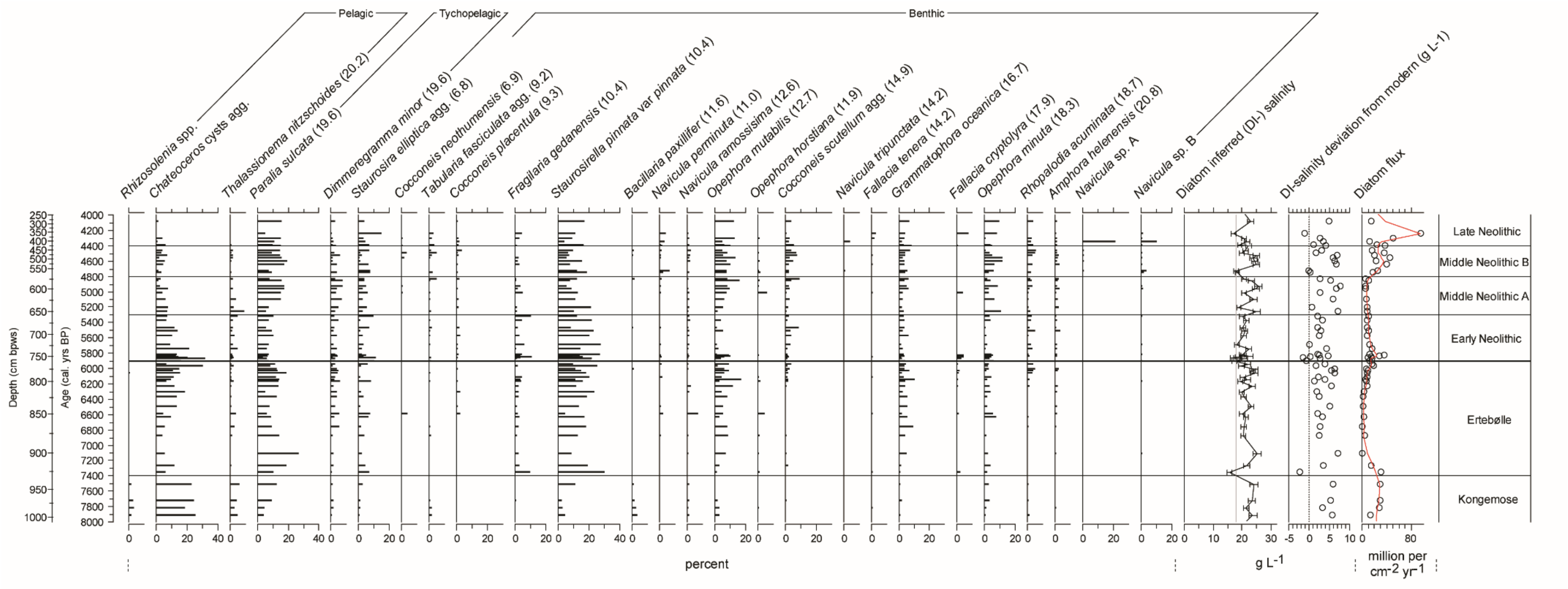

Supplementary Figure 8 I Norsminde Fjord diatom assemblage (selected species > 3%). Associated metrics include diatom-inferred (DI-)salinity (with sample-specific standard errors (via bootstrapping), based on a pan-Baltic 210 site training set; Supplementary Notes 1), DI-inferred salinity deviation from modern (~18 g L^{-1}[118]) and diatom flux (with loess 0.1 span smoother). Diatom data expressed as percentages of the whole assemblage. The diatom assemblage has been ordered via basic habitat type (i.e. pelagic, tychopelagic or benthic) and within these classes via weighted-averaging salinity optima (i.e. values in brackets in g L^{-1}) from fresh/brackish (on left) to marine (on right) generated from the modern diatom training set (Supplementary Notes 1). Sub-divisions follow archaeological periods in Fischer and Kristiansen[115]. Further discussion provided in Lewis[4]. Analyst: Jonathan Lewis

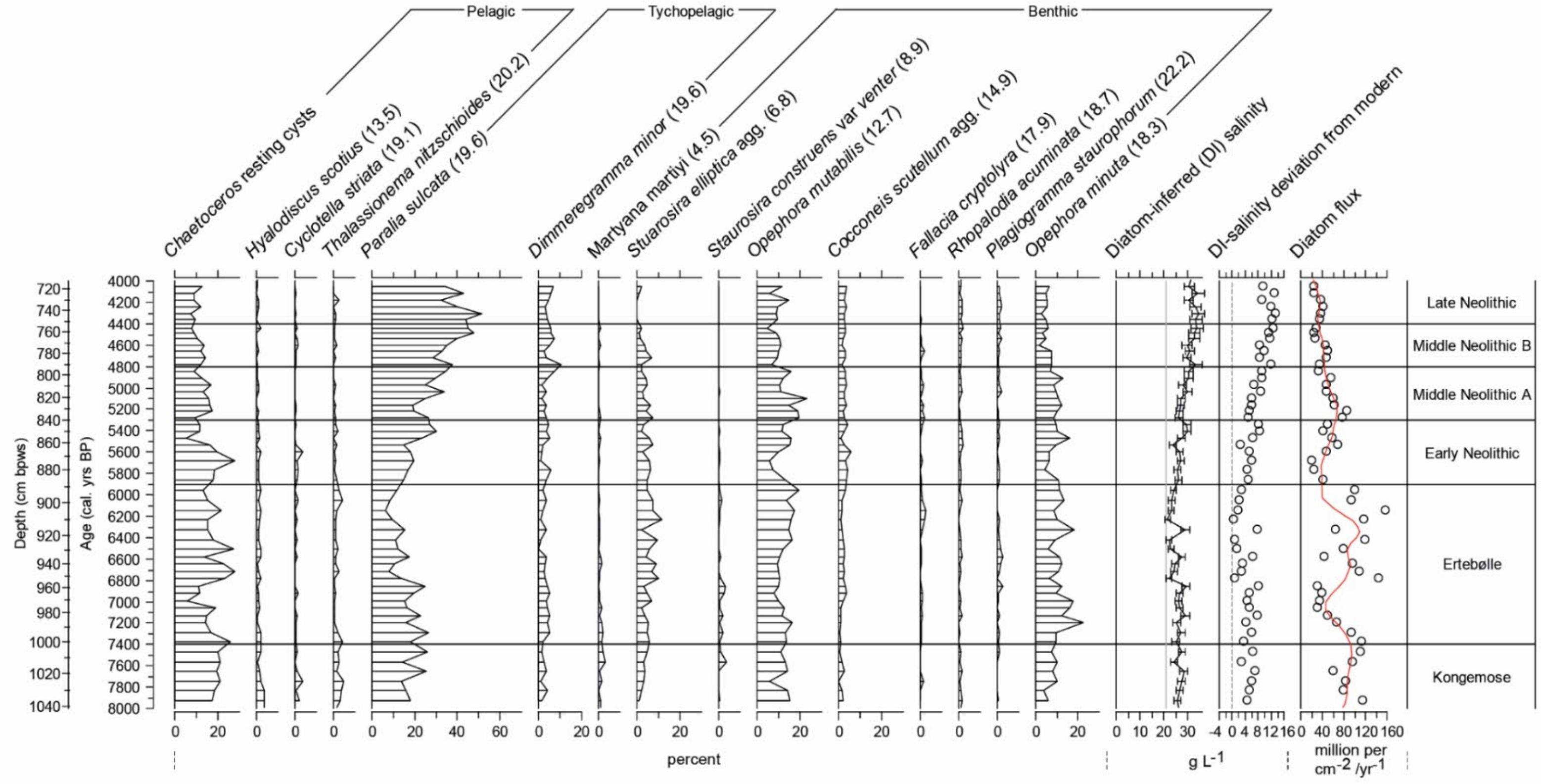

Supplementary Figure 9 I Horsens Fjord diatom assemblage (selected species > 3%). Associated metrics include diatom-inferred (DI-)salinity (with sample-specific standard errors (via bootstrapping) based on a pan-Baltic 210 site training set; Supplementary Notes 1), DI-inferred salinity deviation from modern (21 g L^{-1} [119, 120]) and diatom flux (with loess 0.1 span smoother). Diatom data expressed as percentages of the whole assemblage. The diatom assemblage has been ordered via basic habitat type (i.e. pelagic, tychopelagic or benthic) and within these classes via weighted-averaging salinity optima (i.e. values in brackets in g L^{-1}) from fresh/brackish (on left) to marine (on right) generated from the modern diatom training set (Supplementary Notes 1). Sub-divisions follow archaeological periods in Fischer and Kristiansen[115]. Analyst: Karin Jensen.

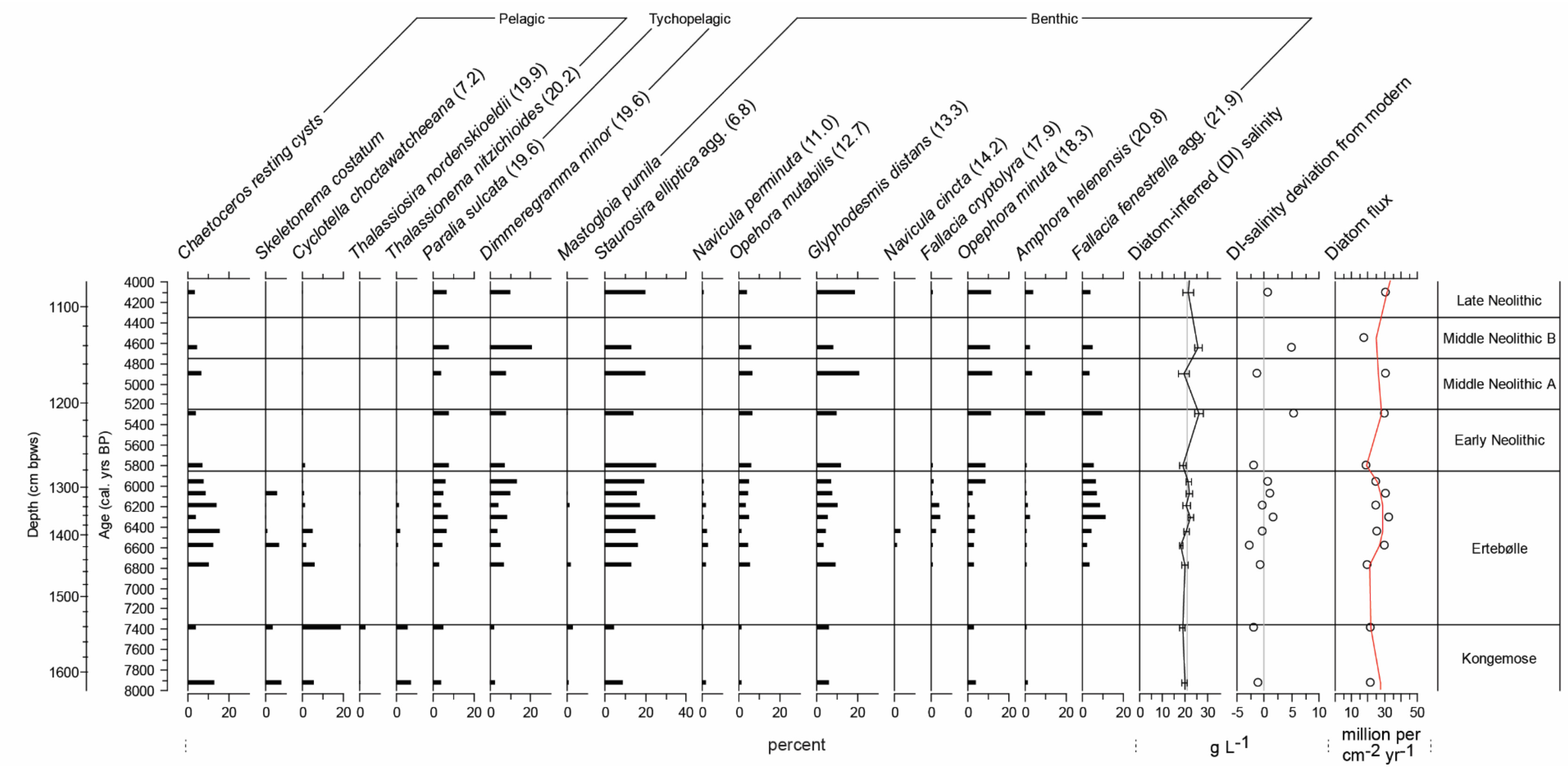

Supplementary Figure 10 I Tempelkrog diatom assemblage (selected species > 3%). Associated metrics include diatom-inferred (DI-)salinity (with sample-specific standard errors (via bootstrapping), based on a pan-Baltic 210 site training set; Supplementary Notes 1), DI-inferred salinity deviation from modern (19.4 g L^{-1} for Inderbredning/Isefjord[119]) and diatom flux (with loess 0.1 span smoother). Diatom data expressed as percentages of the whole assemblage. The diatom assemblage has been ordered via basic habitat type (i.e. pelagic, tychopelagic or benthic) and within these classes via weighted-averaging salinity optima (i.e. values in brackets in g L^{-1}) from fresh/brackish (on left) to marine (on right) generated from the modern diatom training set (Supplementary Notes 1). Sub-divisions follow archaeological periods in Fischer and Kristiansen[115]. Analyst: Annemarie Clarke.

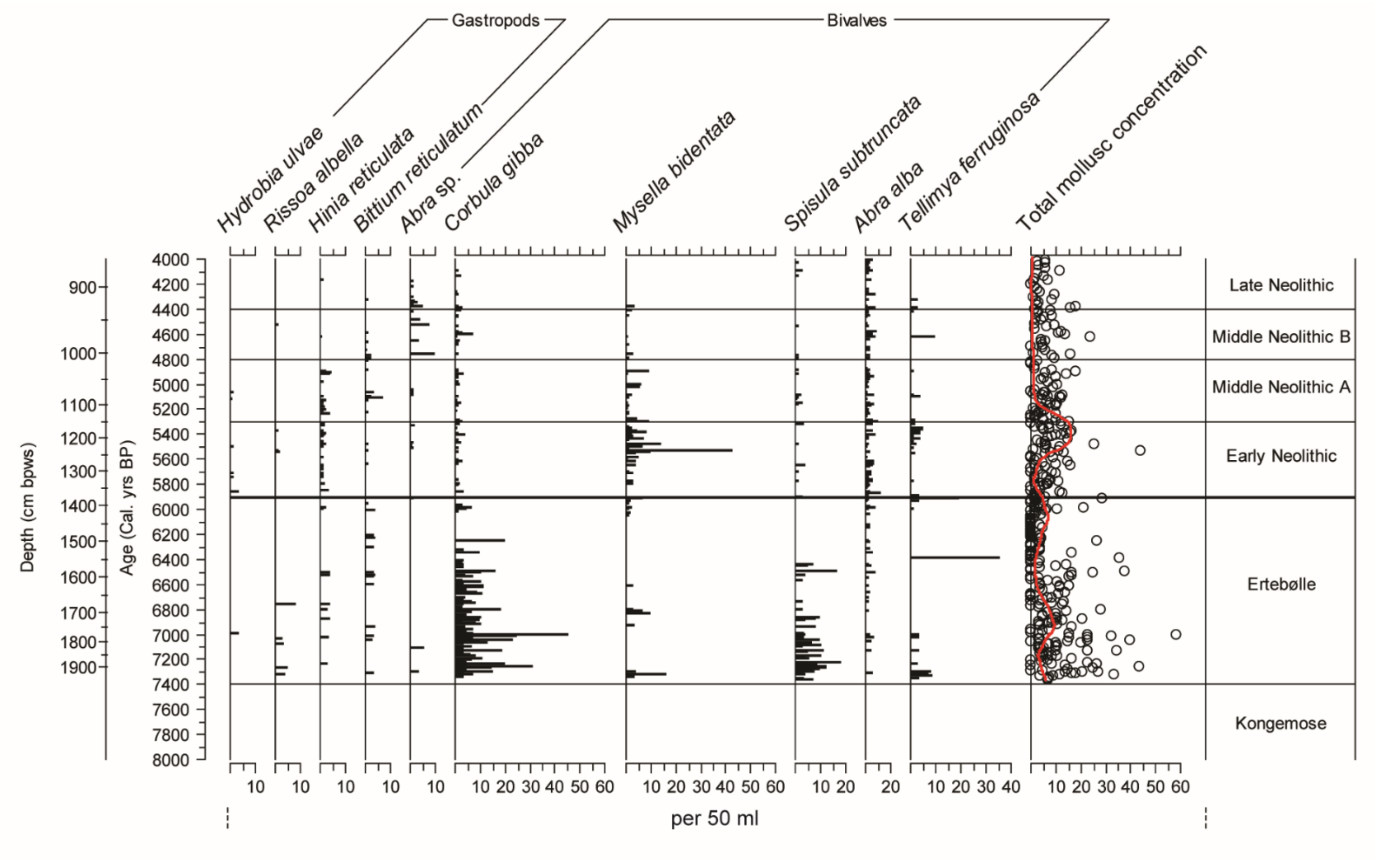

Supplementary Figure 11 I Kilen mollusc assemblage. Selected species and total concentration (with loess smoother (red line); 0.1 span) expressed as concentrations per 50 ml of wet sediment. Molluscs ordered via class (bivalvia or gastropoda) and within these classes via minimum salinity tolerance based on data in Sorgenfrei[121]. All molluscs present in wet sieved fractions (500 and 100 μm) from each core slice were picked and where possible identified to species level. Molluscan nomenclature follows Petersen[122] Sub-divisions follow archaeological periods in Fischer and Kristiansen[115]. Further discussion provided in Lewis; Lewis et al.[4, 19].

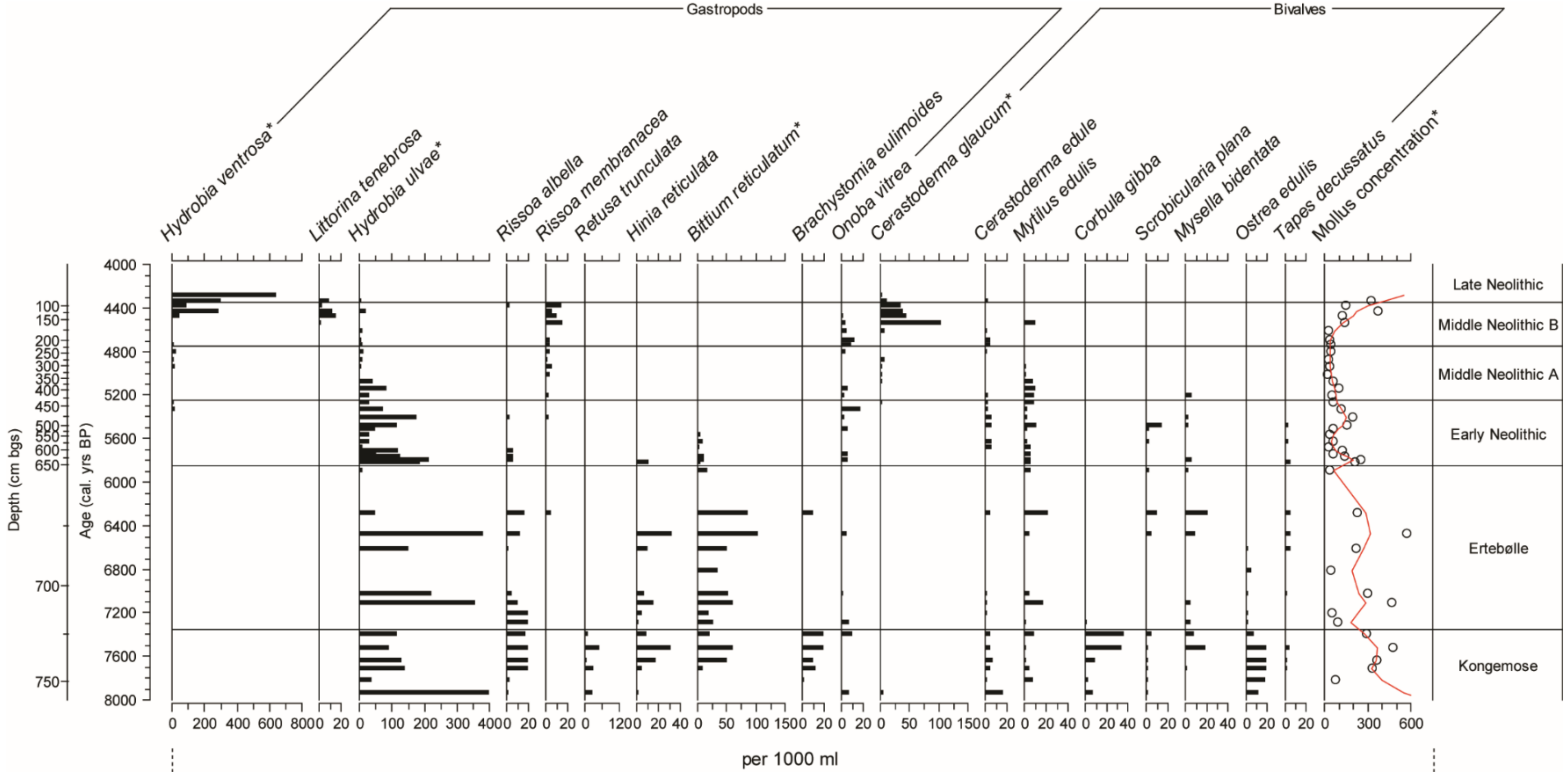

Supplementary Figure 12 I Korup Sø mollusc assemblage. Selected species and total mollusc concentration (with loess smoother (red line); 0.1 span) expressed as concentrations per 1000 ml of wet sediment.. *[asterix] denotes that the plot is displayed on independent scaling to other like plots to improve visual display. Molluscs ordered via class (bivalvia or gastropoda) and within these classes via minimum salinity tolerance based on data in Sorgenfrei[121]. All molluscs present in wet sieved fractions (500 and 100 µm) from each core slice were picked and where possible identified to species level. Molluscan nomenclature follows Petersen[122]. Sub-divisions follow archaeological periods in Fischer and Kristiansen[115]. Further discussion provided in Lewis[4] and Petersen[9]. Analyst: Kaj Strand Petersen.

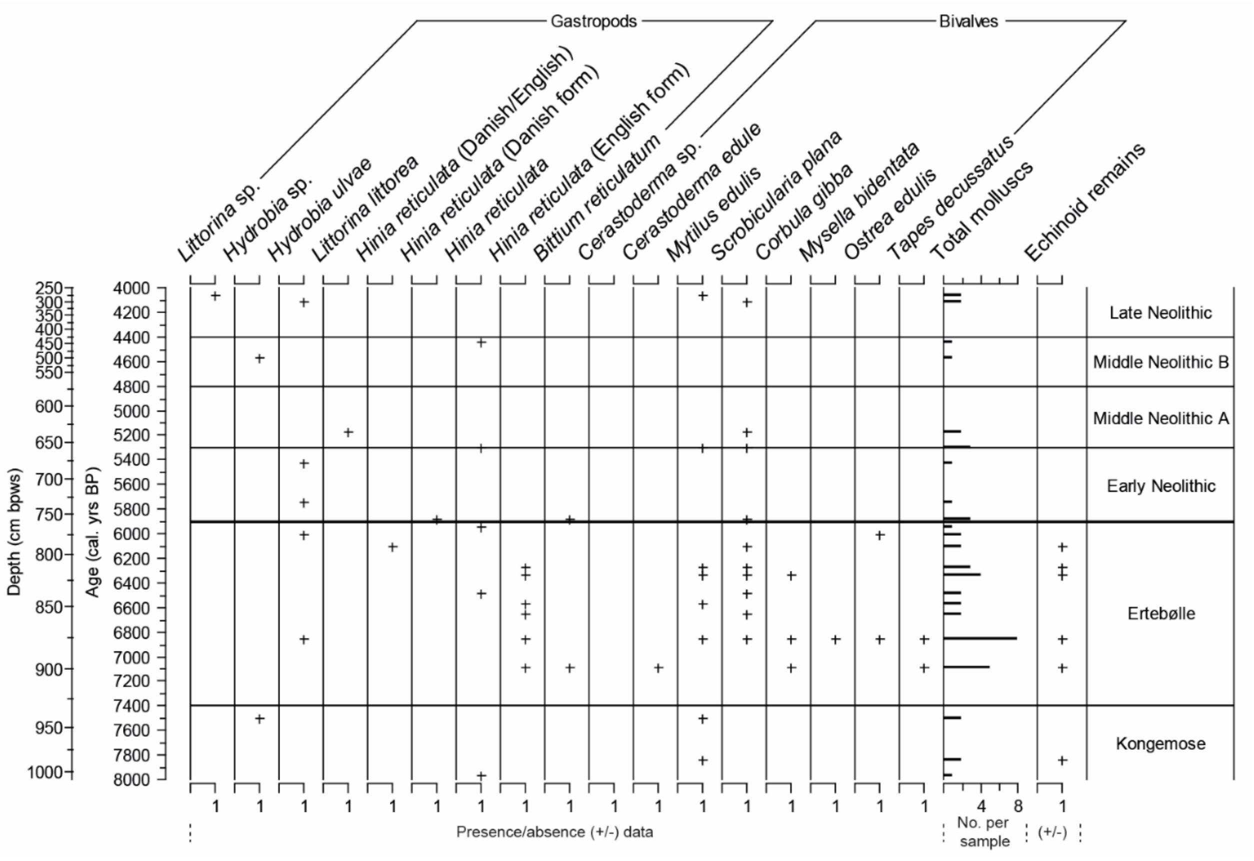

Supplementary Figure 13 I Norsminde Fjord mollusc assemblage. Molluscs expressed as presence/absence data due to the low numbers of molluscs present in the profile. Molluscs ordered via class (bivalvia or gastropoda) and within these classes via minimum salinity tolerance based on data in Sorgenfrei[121]. All molluscs present in wet sieved fractions (500 and 100 µm) from each core slice were picked and where possible identified to species level. Molluscan nomenclature follows Petersen[122]. Remains of echinoids were also recorded,but were unable to be identified to any higher taxonomic level and therefore are also expressed as presence/absence data. Sub-divisions follow archaeological periods in Fischer and Kristiansen[115]. Further discussion provided in Lewis[4]. Analyst: Kaj Strand Petersen.

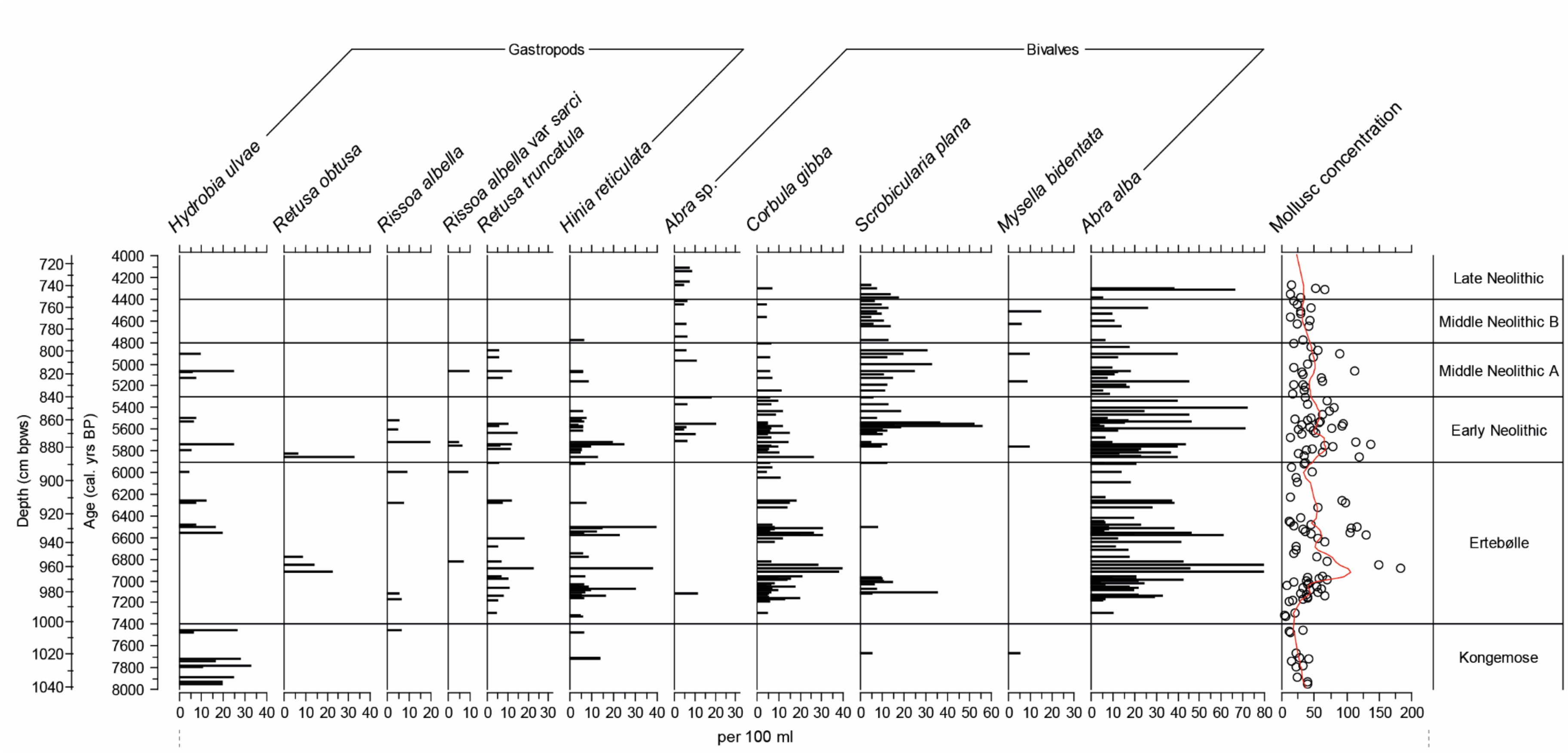

Supplementary Figure 14 I Horsens Fjord mollusc assemblage. Selected species and total mollusc concentration (with loess smoother (red line); 0.1 span) expressed as concentrations per 100 ml of wet sediment. Molluscs ordered via class (bivalvia or gastropoda) and within these classes via minimum salinity tolerance based on data in Sorgenfrei[121]. All molluscs present in wet sieved fractions (500 and 100 μm) from each core slice were picked and where possible identified to species level. Molluscan nomenclature follows Petersen[122]. Sub-divisions follow archaeological periods in Fischer and Kristiansen[115]. Analyst: Kaj Strand Petersen.

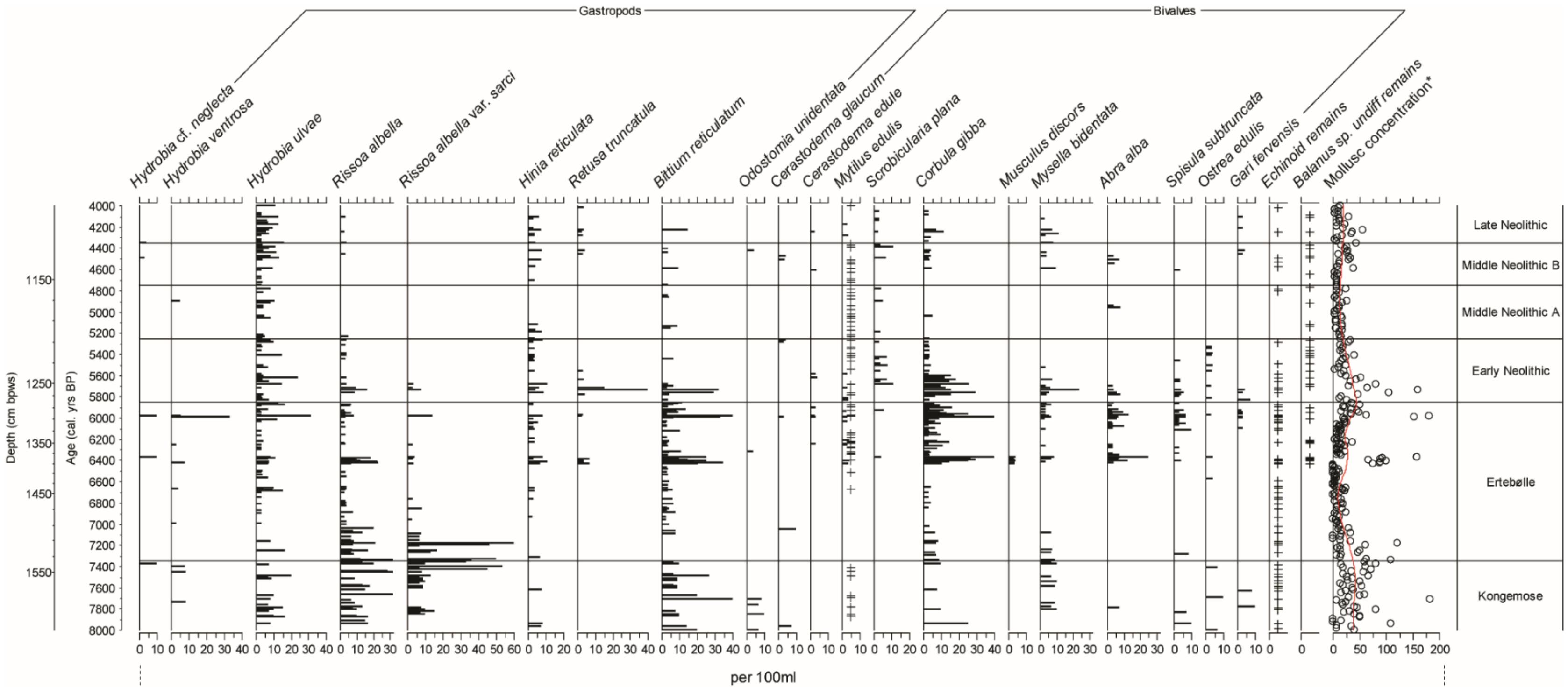

Supplementary Figure 15 I Tempelkrog mollusc assemblage. Selected species and total mollusc concentration (with loess smoother (red line); 0.1 span) expressed as concentrations per 100 ml of wet sediment.. Molluscs ordered via class (bivalvia or gastropoda) and within these classes via minimum salinity tolerance based on data in Sorgenfrei[121]. All molluscs present in wet sieved fractions (500 and 100 µm) from each core slice were picked and where possible identified to species level. Molluscan nomenclature follows Petersen[122] Remains of echinoids and barnacles (*Balanus* sp.) were also recorded, but were unable to be identified to any higher taxonomic level and therefore are also expressed as presence/absence data. Sub-divisions follow archaeological periods in Fischer and Kristiansen[115]. Analyst: Kaj Strand Petersen.

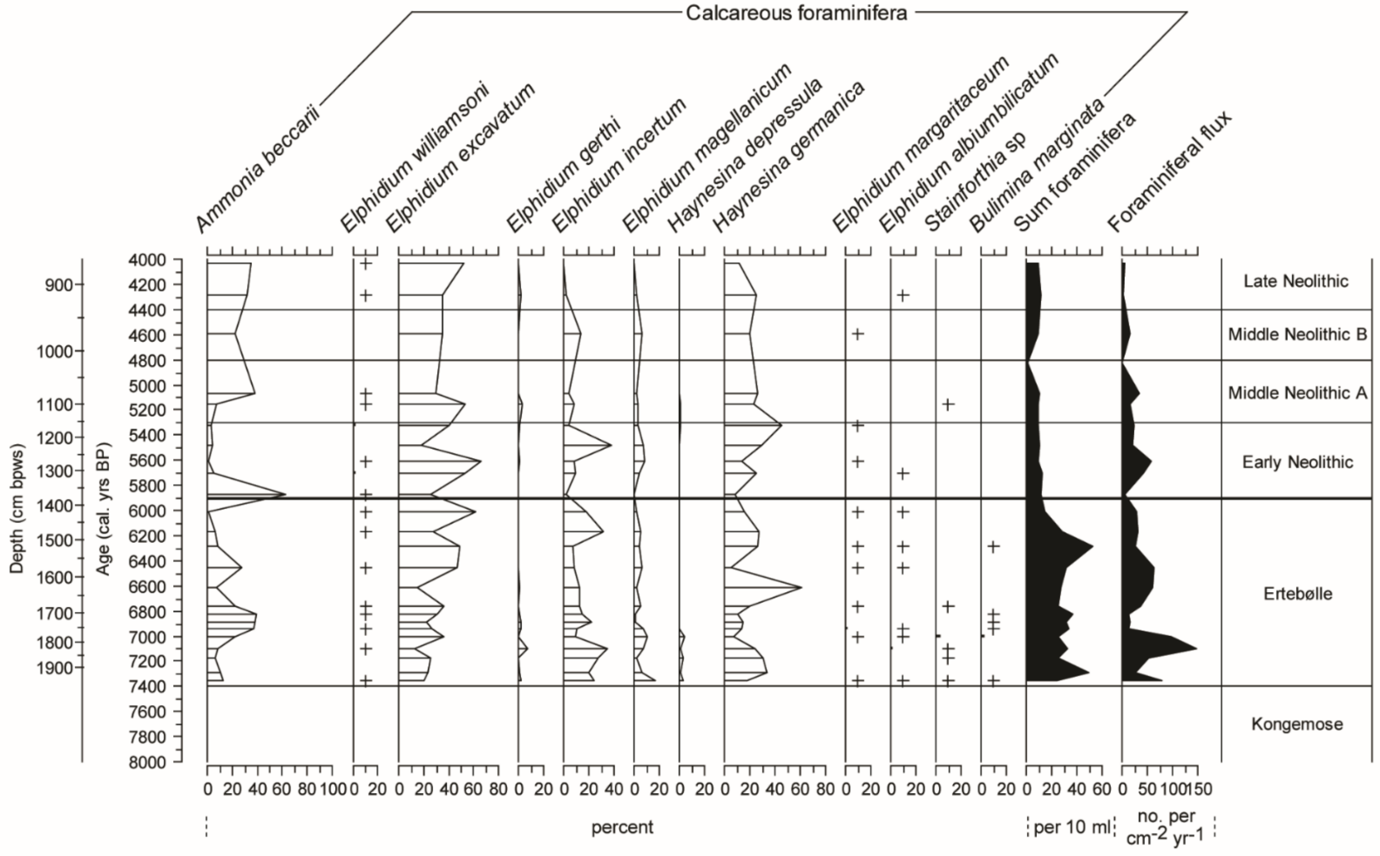

Supplementary Figure 16 I Kilen foraminiferal assemblage. Data expressed as percentage of the whole calcareous foraminiferal assemblage. In each sample, rare taxa (below 1% cut off) are displayed with a '+' to indicate presence. Taxonomy and nomenclature follows Ellis and Messina[123]. Sub-divisions follow archaeological periods in Fischer and Kristiansen[115]. Further discussion provided in Lewis, Lewis et al.[4, 19]. Analyst: Karen Luise Knudsen.

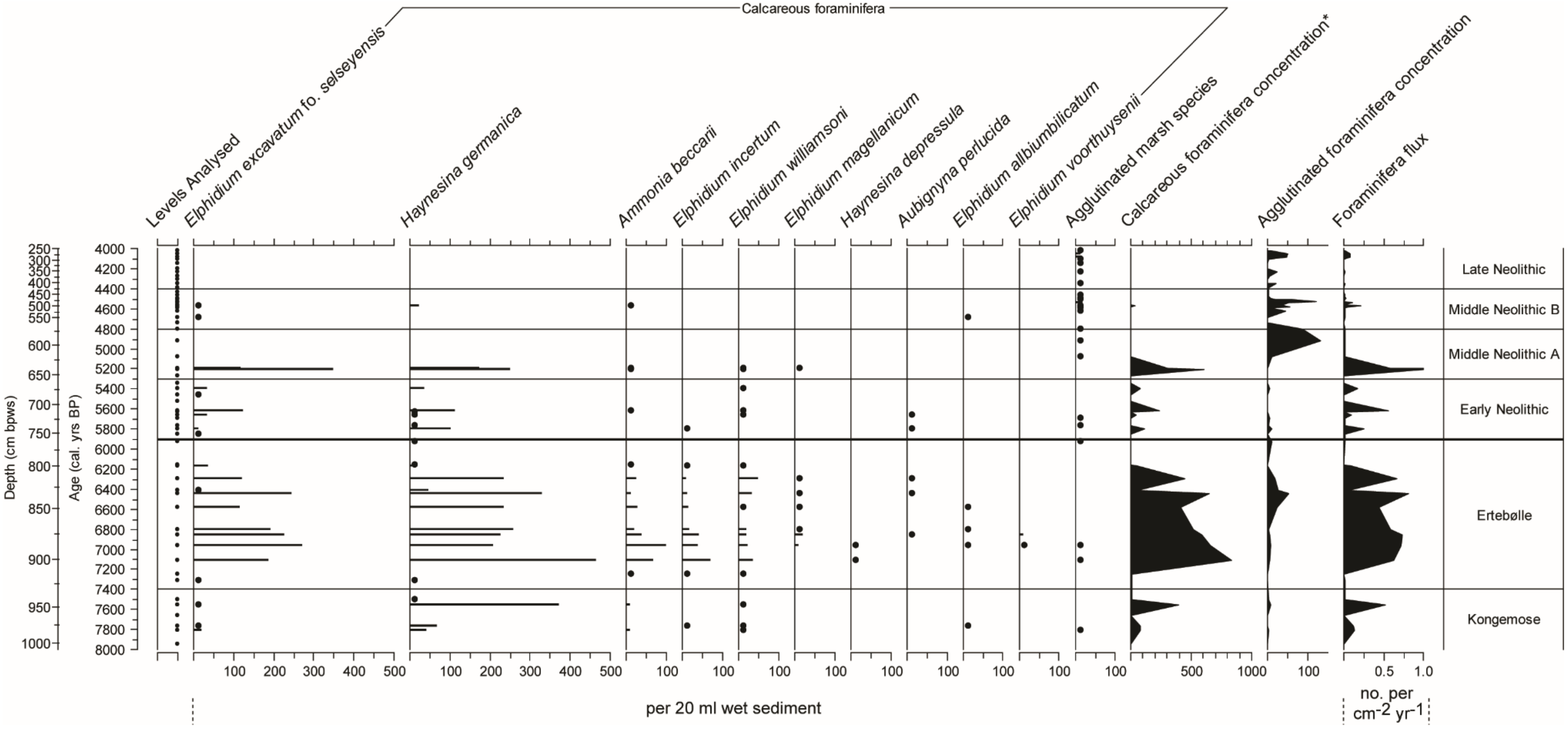

Supplementary Figure 17 I Norsminde foraminiferal assemblage. Data expressed as concentration per 20 ml of wet sediment due to variable abundances. A column indicating all level analysed has been included to demonstrate the paucity of the foraminiferal data. In each sample, rare taxa are indicated with a (dot) symbol (i.e. taxa with concentration <10 per 20 ml). Taxonomy and nomenclature follows Ellis and Messina[123]. Sub-divisions follow archaeological periods in Fischer and Kristiansen[115]. Further discussion provided in Lewis[4]. Analysts: Peter Konradi and Karen Luise Knudsen.

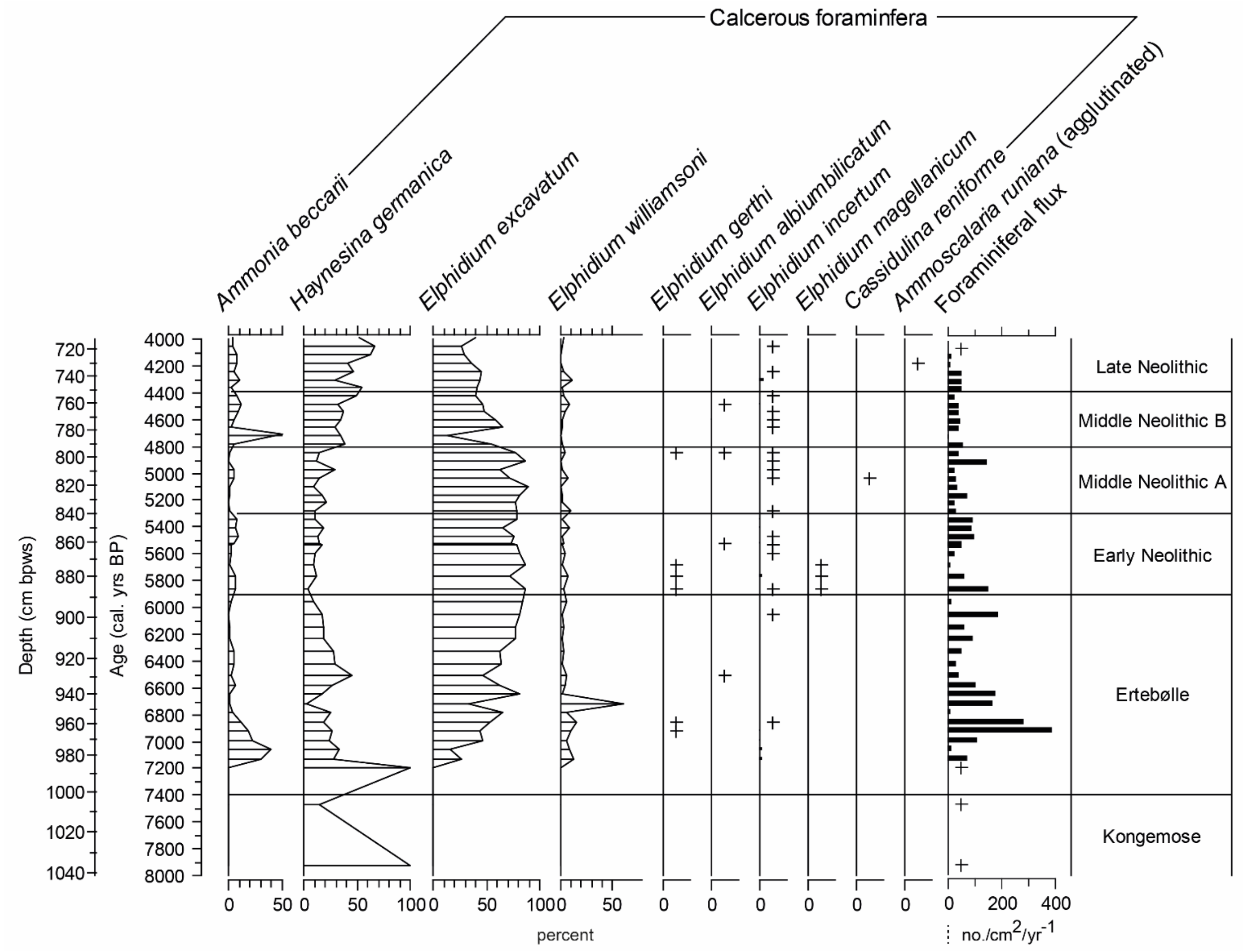

Supplementary Figure 18 I Horsens foraminiferal assemblage. Data expressed as percentages of the total calcareous foraminiferal assemblage. In each sample rare taxa (below 1% cut off) are displayed with a '+' to indicate presence. Taxonomy and nomenclature follows Ellis and Messina[123]. Sub-divisions follow archaeological periods in Fischer and Kristiansen[115]. Analyst: Karen Luise Knudsen.

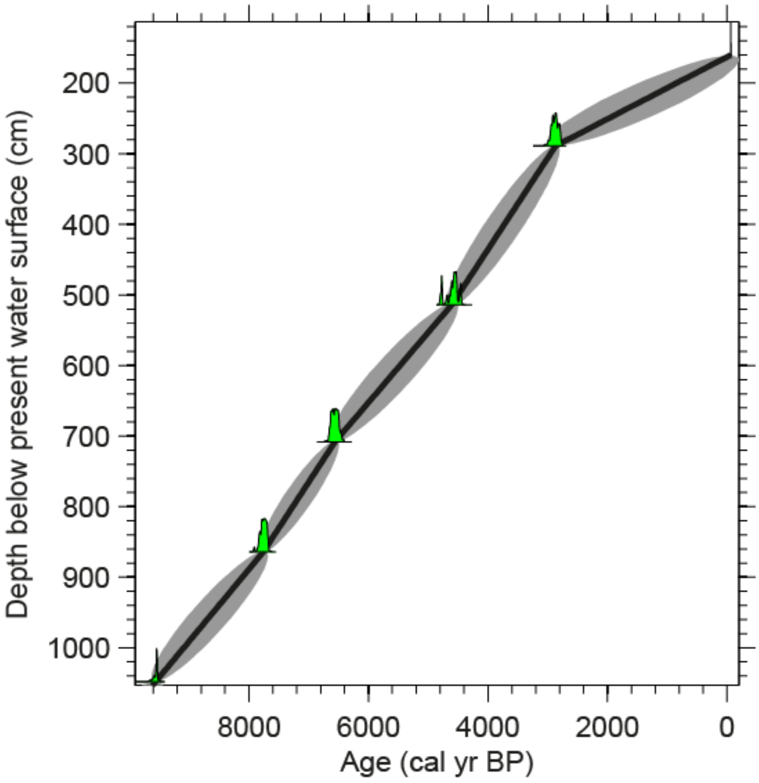

Supplementary Figure 19 I Age-depth model for Sebbersund. Model based on 4 dates derived from terrestrial material (Supplementary Table 3) and one bulk peat sample collected from the lowermost limnic part of the sequence, prior to the marine transgression. An additional terrestrially-derived AMS ^{14}C date was omitted form the sequence due to its poor fit (several thousand years too old; Supplementary Table 3). This age-depth model was produced using Oxcal 4.3[11] with the atmospheric calibration curve IntCal13[8].

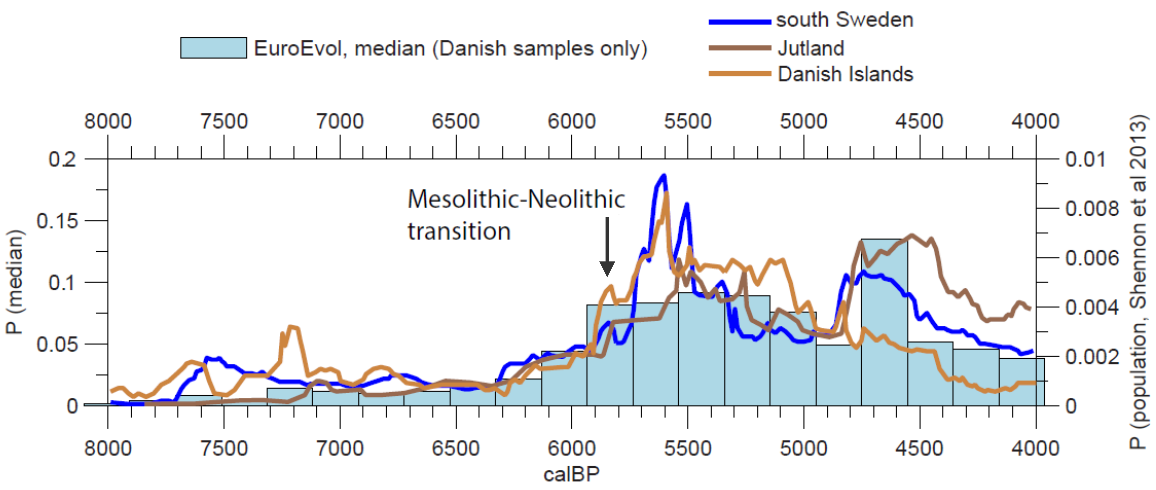

Supplementary Figure 20 I Population change metrics over the study period. Eurovol database[53] ^{14}C sample medians for Denmark plotted against population densities for Jutland, Danish Islands and south Sweden in Shennan et al.[33] using the summed probability distribution (SPD) method.

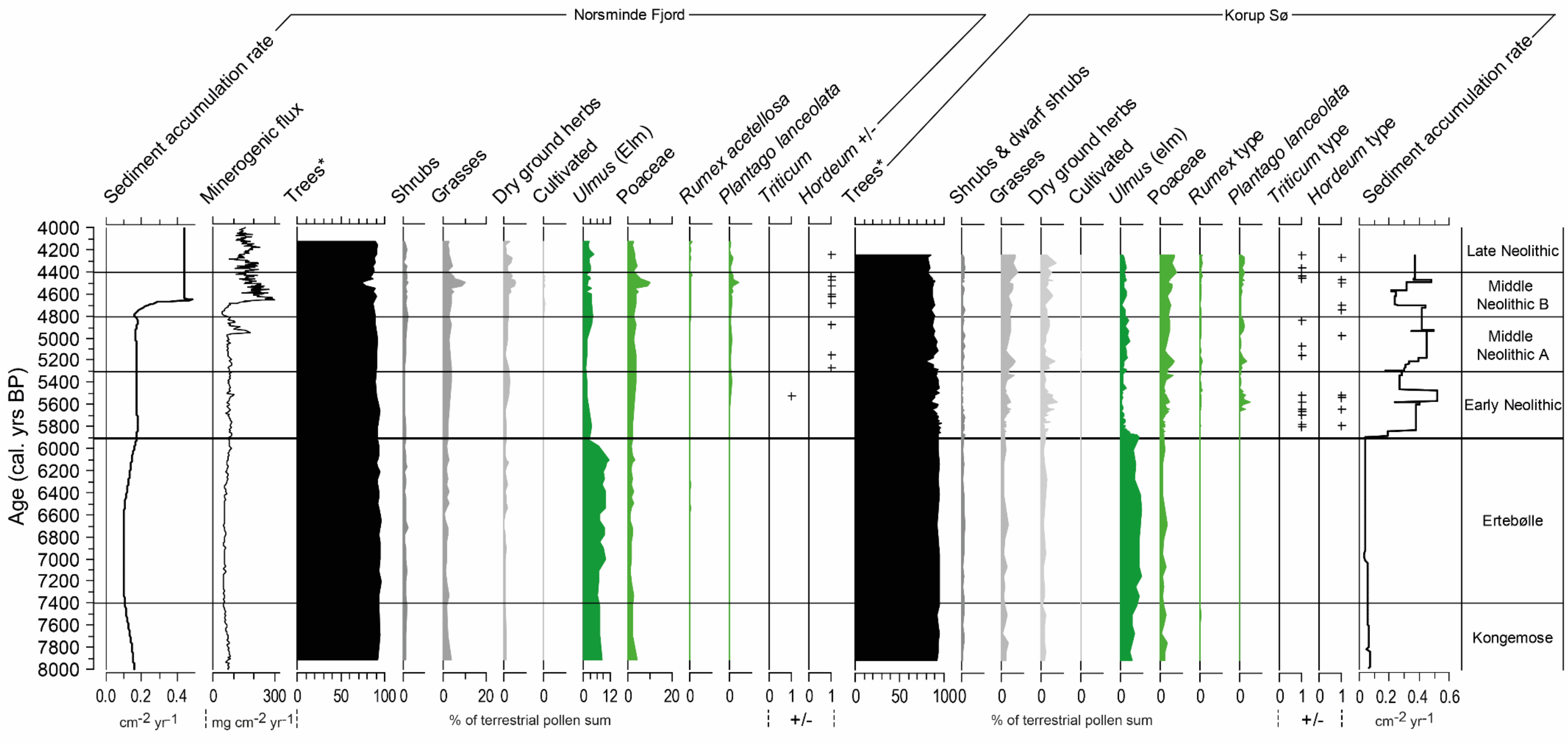

Supplementary Figure 21 I Pollen indicators of anthropogenic disturbance. Selected pollen taxa from two coastal sites, Korup Sø (unpublished data; Harald Krog) and Norsminde Fjord (unpublished data; Peter Rasmussen), indicative of human disturbance of the catchment and/or agricultural activity. Note the general stability of the sediment accumulation rate (and minerogenic curve for Norsminde Fjord) in the pre-agricultural era, in comparison to the Neolithic period, when humans increasingly manipulate the catchment for agricultural purposes. *Trees on independent scaling to other pollen taxa. Sub-divisions follow archaeological periods in Fischer and Kristiansen[115].

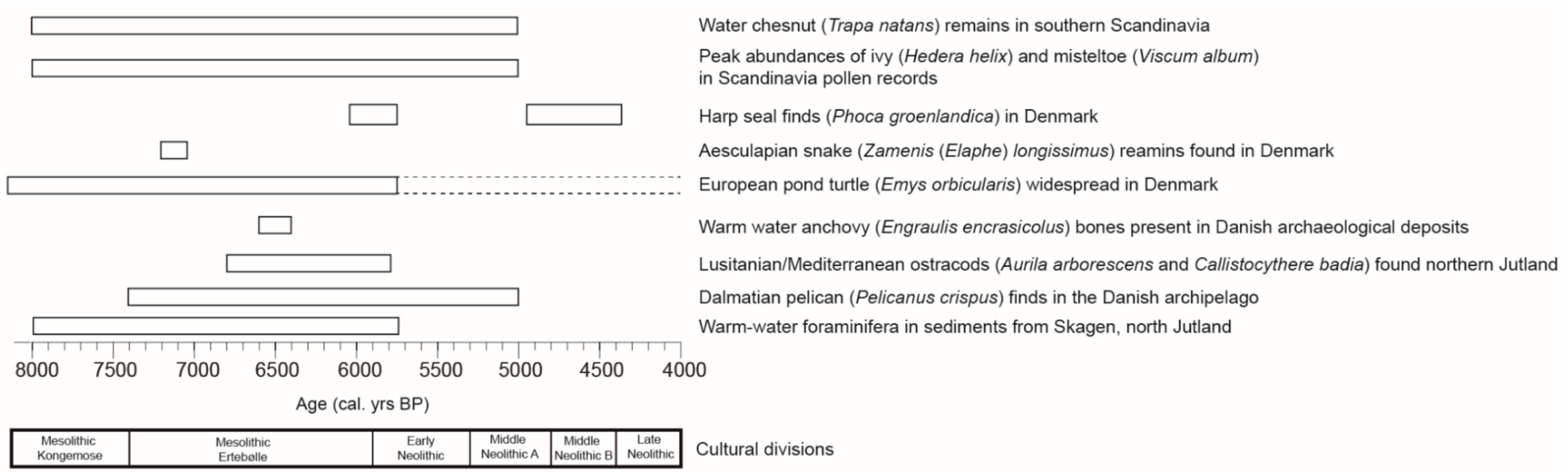

Supplementary Figure 22 I Key Holocene thermal maximum (HTM) indicator species in Southern Scandinavia. Presence of indicator species of high temperatures and/or more oceanic conditions (than present day) existing during the HTM (8000-4000 BP; peak temperature 7500-6000 BP[81, 82, 83]) in south Scandinavia and the surrounding coastal/oceanic waters (including North Sea, Skagerrak, Kattegat, and Baltic Sea). Data sources: Presence of water chesnust (*Trapa natans*) in sediments from east Danish lakes and frequent presence of ivy and misteltoe in Scandinavian pollen diagrams, considered classic indicators of higher temperature during the mid-Holocene thermal maximum[73, 85]. Harp seal finds in Danish waters indicate a suitable habitat for this species in south Scandiavia waters (i.e. higher salinity and marine productivity than present day) during the mid-Holocene[124]. High-temperature demanding Aesculapian snake remains present in a Danish Mesolithic settlement[91]. This species current range is restricted to southern Europe. European Pond turtle present in Danish waters coinciding with the highest temperatures of the Holocene thermal maxium[90]. After ca. 5800 BP, the northern population collapsed (dotted line) likely due to declining temperatures in the later part of the Holocene Thermal Maximum. Bones of the anchovy *Engraulis encrasicolus* present in a shell midden from Krabbesholm, in the Limfjord[46]. Sub-fossil remains of Lusitanian/Mediterranean ostracod species (currently absent) from Danish waters from several deposits in north Jutland, Denmark indicative of higher sea-surface temperatures during the mid-Holocene[89]. Presence of

Dalmatian pelican remains in north-central European (including Denmark) archaeological/geological deposits during the mid-Holocene, far out of its present day range in south east Europe[125]. High-temperature demanding foraminfiera (e.g. *Uvigerina mediterranea*) present in a core from Skagen in northern Jutland[126]. Sub-divisions follow archaeological periods in Fischer and Kristiansen[115].

Supplementary Tables

**Site (coring summary) and references containing published methodology/data from site:**	**Description/details**
**Kilen:** Two closely spaced boreholes were cored (in 2007) using Russian augers (10 cm and 5 cm diameter; 100 cm chamber length), with overlapping sections (of 50 cm). The overlapping sequences were correlated where possible by physical parameters* (subsampled at 1 or 2 cm resolution). **Key References:** Lewis[4]; Philippsen et al.[6]; Lewis et al. [14, 19]	Kilen is a west to east facing fjord situated in the western Limfjord (north Jutland) near Struer and today, almost entirely isolated from the Limfjord by a road and rail embankment (completed in AD 1856). A narrow connection with the Limfjord is retained via a small stream (termed the 'Kilerkanal') in the south-east corner. Modern average salinity within the fjord is ~8 g L^{-1}, but in the adjacent Struer Bay, salinities are 24-27 g L^{-1}. Maximum depth = 6.5 m, average depth = 2.5m with a surface area of ~3.3 km^2 and draining a catchment area of ~35.3 km^2.
**Korup Sø:** Two boreholes (Well no. 1 and 2) were drilled in 1982/83 (Petersen[9]). All analyses presented here have been performed on material from Well No. 1, with a maximum depth of 880 cm. Core correlation was not attempted between the parallel drillings due to the significant distance between boreholes (~300 m) and the absence of physical parameters. **Key References:** Petersen[9]; Lewis[4]; Lewis et al.[14]	Korup Sø is situated in the central part of the Djursland peninsula (today dry land, ~3 m above sea level) northeast of the city of Aarhus in east Jutland[4]. Due to sea level decline and sediment accumulation the fjord became isolated from the Kolindsund-Randers fjord system several thousand years ago[4, 9].
**Norsminde Fjord:** Cored (in 1996) using Russian auger (with diameters of 10, 7.5 and 5 cm; all with 100 cm chamber lengths) from 2 boreholes (1 m apart), with overlapping sections (of 20 cm), correlated by physical parameters (particularly minerogenic residue (following ignition) at 2 cm subsampling resolution. **Key References:** Lewis[4]; Lewis et al.[14]	Elongated southwest to northeast facing fjord basin situated at the east coast of Jutland about 20 km south of the city of Aarhus. A narrow artificially maintained entrance opens into the western Kattegat. Modern average salinity = 18-20 g L^{-1}. Maximum depth = 2 m, average depth = 0.6 m with a surface area of ~1.68 km^2 and draining a catchment area of ~101 km^2.
**Horsens Fjord:** Cored (in 2004) using Russian augers (with diameters of 10, 7.5 and 5 cm; all 100 cm chamber lengths) from 2 closely spaced boreholes with overlapping sections (of 50 cm) between drives. Overlapping section where correlated using physical analyses, at 1 cm subsampling resolution. **Key References**: Rasmussen et al.[127]; Olsen et al.[5]; Lewis et al.[14]	Horsens Fjord is a relatively large, west to east facing shallow fjord, situated on the east coast of Jutland (~20 km further south of Norsminde Fjord). Horsens fjord has a broad opening (~4-5 km wide) into the western Kattegat, just north of the Danish Belt Sea. Modern average salinity = 21 g L^{-1}. Maximum depth = 22 m, average depth = 2.9m with a surface area of ~77.5 km^2 and draining a catchment area of ~449 km^2.
**Tempelkrog:** Cored (in 2005) using Russian augers (with diameters of 10, 7.5 and 5 cm; all 100 cm chamber lengths) from 2 closely spaced boreholes with overlapping sections (of 50 cm) between drives. Overlapping sections where	Tempelkrog is a small, north facing, shallow basin situated at the southern end of Isefjord in the north of Zealand. Tempelkrog in one of several branches (including Roskilde Fjord, Lammefjord, Holbæk fjord) connecting to the Isefjord, which open into the southern Kattegat.

correlated via physical analyses, at 2 cm subsampling resolution. **Key References**: Rasmussen et al.[127]; Olsen et al.[5]; Lewis et al.[14]	Modern average salinity of the wider Isefjord = 19.4 g L^{-1}. Maximum depth = 17 m, average depth = 4.9 m with a surface area of ~307 km^2 and draining a catchment area of ~776 km^2.
**Sebbersund:** Two closely spaced boreholes were cored (in 2014) using Russian auger (chamber 10 cm in diameter and 100 cm long). Overlapping sections of 50 cm between boreholes were correlated using physical parameters* (at 1 or 2 cm subsampling resolution). **Key references:** No published data	Sebbersund is a small, shallow (<2.5 m deep) coastal basin connecting the fjord site of Halkær Bredning to Nibe Bredning in the north east Limfjord. From here the Limfjord has both a narrow connection to the central Limfjord to the west (through the Aggersund) and to the Kattegat in the east through the Langerak. Modern average salinity of the Nibe Bredning is ~25 g L^{-1}; ~20 g L^{-1} at Sebber). Maximum depth of Sebbersund ~2.4 m, average depth = 1-2 m.

Supplementary Table 1 I Details of the six Danish coastal sites analysed for palaeoenvironmental change. More detailed site descriptions can be found in the references. In all cases except Korup Sø, the uppermost sediments were collected via a HON-Kajak corer[128], but these most recent sediments are outside the scope of this study. *Here, physical parameters refer to lithology, organic matter content, calcium carbonate ($CaCO_3$) content and remaining minerogenic residue.

Site	No of ^{14}C dates	Record span	Material	Age-depth model	Age-model details
Sebbersund	5	Last ~9000 years	Terrestrial plant macrofossils	Oxcal	G. Weiss, J.P. Lewis et al. unpub. data
Kilen	19	Last ca. 7500 years	Terrestrial plant macrofossils*	Oxcal	Lewis[4]; Philippsen et al.[6]
Korup Sø	8	Ca. 9600-4200 BP	All shells with (400 year) correction for marine reservoir effect*.	Oxcal	Petersen[9]; Lewis[4]
Norsminde Fjord	14	9000-3700 BP	Terrestrial macrofossils*	B-peat	Lewis[4]
Horsens Fjord	12	Last ~9000 years	Terrestrial macrofossils	Oxcal	Olsen et al.[5]
Tempelkrog	13	Last ~9000 years	Terrestrial macrofossils	Oxcal	Olsen et al.[5]

Supplementary Table 2 I Sedimentary sequences and chronological details for the six Danish coastal sites presented here. * The age of the Ulmus (elm) decline (pollen analysis) has been used to verify the chronology.

Laboratory No.	Sequence depth (cm)	Material dated	^{14}C age (^{14}C yrs BP)
UBA-27257	284-294	13 wood fragments. Weight 0.8mg	2778±47
UBA-26961	513-515	12 twigs/wood fragments (with bark) and 3 deciduous leaf fragments. Weight: 2.8 mg	4077±36
UBA-26271	707-709	Several pieces of bark (possibly *Betula*) Weight: 7.4 mg	5761±44
UBA-26272**	817-819	45 deciduous leaf fragments. Weight: 2 mg	8064±44
UBA-26960	863-865	1 Betula fruit, 5 fragments of wood (with bark), 1 twig, 16 deciduous leaf fragments and 8 fragments of bark. Weight: 2.6 mg	6925±44
UBA-26273	1047-1049	bulk peat	8596±38

Supplementary Table 3 I Radiocarbon ages of terrestrial plant material from Sebbersund. **Date omitted from the age-depth model due to poor fit (clear evidence of an age reversal).

Region/time interval	Pop. 1/pre-Pop. 1	Pop. 2/pre-Pop. 2
Time intervals compared (BP)	7600-7100 : 8100-7600	6400-5900 : 6900-6400
Scania	<0.0001	<0.0001**
Jutland	0.027199**	0.001008
Danish Islands	0.001273**	0.047185**

Supplementary Table 4 I Population statistical test results. p-values of one-tailed t-tests between Summed Probability Distribution (SPD) values for each region between population increase 1 (Pop. 1, 7600-7100 BP) and population increase 2 (Pop. 2, 6400-5900 BP) and preceding 500 years. ** samples with unequal variances.

Technology	Use	First appearance
**Marine:**		
Fish trap	Stationary fishing trap, held in place by vertical wooden stakes or large stones (often found near/in front of marine sites). Two types: round opening fish trap and semi-circular/rectangle opening.	Early Kongemose
Lance	Pointed bone or antler. Oval or round in cross section. Likely used similar to harpoons for sea mammal hunting.	Early Ertebølle
Dugout canoe	Early boat carved from wooden trunks for sea travel (and social contact along coasts) and fishing (including sea mammals).	Early Ertebølle
Paddles	Carved from wood to paddle dugout canoes.	Early Ertebølle
Fish hook	Made from wood, bone and antler for line fishing (demonstrating multiple methods of fishing in this period). Ertebølle fish hooks usually 2-3 cm in length (with no barb). Found from many sites across Denmark with some regional variations (and likely uses).	Middle Ertebølle
Leister	A type of pronged spear made from wood to catch fish (likely used in eel fishing). Two different types; short stubby and long and slender varieties, the former used in 'hard' substrate and the latter in 'softer' substrates.	Middle Ertebølle
Harpoon	Made of antler or whale bone, harpoons were likely used in seal/whale hunting at sea.	Middle Ertebølle
Fish net	Made from plant fibres. Associated floats made from wood and stones as sinkers for these nets found at multiple sites from the Late Ertebølle.	Late Ertebølle
**Terrestrial:**		
Polished flint axe	Wooden (ash) axe helve with polished flint axe - a paramount tool behind expansion of first farmers. Used for a variety of tasks including felling trees and timber work.	Early Neolithic ~5900 BP (3900 BC)
Ard	A primitive plough enabling shallow ploughing of the land. Made of wood and had no mouldboard so only scraped furrows in the soil without turning it over.	Early to Middle-Neoltihic; ~5500 BP (3500 cal. yrs BC)

Supplementary Table 5 I Summary of key marine and terrestrial technologies discovered over the study period ca. 8000-4000 BP. See Andersen[63], Thrane[66], Andersen[65], Kristiansen[67], Pedersen[129], Pickard and Bonsall[130] and references therein for more detailed reviews.

Supplementary References

1. Rasmussen P, Pantopoulos G, Jensen JB, Olsen J, Røy H, Bennike O. Holocene sedimentary and environmental development of Aarhus Bay, Denmark – a multi-proxy study. *Boreas* **49**, 108-128 (2019).

2. Dean WE. Determination of Carbonate and Organic-Matter in Calcareous Sediments and Sedimentary-Rocks by Loss on Ignition - Comparison with Other Methods. *Journal of Sedimentary Petrology* **44**, 242-248 (1974).

3. Heiri O, Lotter AF, Lemcke G. Loss on ignition as a method for estimating organic and carbonate content in sediments: reproducibility and comparability of results. *Journal of Paleolimnology* **25**, 101–110 (2001).

4. Lewis JP. Holocene environmental change in coastal Denmark: interactions between land, sea and society. In: *Department of Geography*). Loughborough University (2011).

5. Olsen J, Rasmussen P, Heinemeier J. Holocene temporal and spatial variation in the radiocarbon reservoir age of three Danish fjords. *Boreas* **38**, 458-470 (2009).

6. Philippsen B, Olsen J, Lewis JP, Rasmussen P, Ryves DB, Knudsen KL. Mid- to late-Holocene reservoir-age variability and isotope-based palaeoenvironmental reconstruction in the Limfjord, Denmark. *The Holocene* **23**, 1017–1027 (2013).

7. Stuiver M, Polach HA. Reporting of C-14 data. *Radiocarbon* **19**, 355-363 (1977).

8. Reimer PJ*, et al.* IntCal13 and Marine13 radiocarbon age calibration curves 0-50,000 years cal BP. *Radiocarbon* **R55**, 1869–1887 (2013).

9. Petersen KS. Environmental changes recorded in the Holocene molluscan faunas from Djursland, Denmark. *Scripta Geologica Special Issue* **2**, 359-369 (1993).

10. Fægri K, Iversen J. *Textbook of pollen analysis*. Munksgård (1975).

11. Bronk Ramsey C. Deposition models for chronological records. *Quaternary Science Reviews* **27**, 42-60 (2008).

12. Bronk Ramsey C. Bayesian analysis of radiocarbon dates. *Radiocarbon* **51**, 337-360 (2009).

13. Blaauw M, Christen JA. Radiocarbon peat chronologies and environmental change. *Applied Statistics* **54**, 805-816 (2005).

14. Lewis JP*, et al.* The shellfish enigma across the Mesolithic-Neolithic transition in southern Scandinavia. *Quaternary Science Reviews* **151**, 315-320 (2016).

15. Battarbee RW*, et al.* Diatoms. In: *Tracking Environmental Change Using Lake Sediments* (eds Smol JP, Birks HJB, Last WM). Kluwer Academic Publishers (2001).

16. Battarbee RW. Diatom analysis. In: *Handbook of Holocene Palaeoecology and palaeohydrology* (ed Berglund BE). John Wiley & Sons (1986).

17. Renberg I. A Procedure for Preparing Large Sets of Diatom Slides from Sediment Cores. *Journal of Paleolimnology* **4**, 87-90 (1990).

18. Battarbee RW, Kneen MJ. The Use of Electronically Counted Microspheres in Absolute Diatom Analysis. *Limnology and Oceanography* **27**, 184-188 (1982).

19. Lewis JP*, et al.* Environmental change in the Limfjord, Denmark (ca 7500–1500 cal yrs BP): a multiproxy study. *Quaternary Science Reviews* **78**, 126-140 (2013).

20. Telford RJ, Birks HJB. The secret assumption of transfer functions: problems with spatial autocorrelation in evaluating model performance. *Quaternary Science Reviews* **24**, 2173-2179 (2005).

21. Telford RJ, Birks HJB. Evaluation of transfer functions in spatially structured environments. *Quaternary Science Reviews* **28**, 1309-1316 (2009).

22. Trachsel M, Telford RJ. Technical Note: Estimating unbiased transfer-function performances in spatially structured environments. . *Climate of the Past* **12**, 1215-1223 (2016).

23. Brock F, Higham T, Ditchfield P, Bronk Ramsey C. Current pretreatment methods for AMS radiocarbon dating at the Oxford Radiocarbon Accelerator Unit (Orau). *Radiocarbon* **52**, 103-112 (2010).

24. Longin R. New method of collagen extraction for radiocarbon dating. *Nature* **230**, 241-242 (1971).

25. Brown TA, Nelson DE, Vogel JS, Southon JR. Improved collagen extraction by modified Longin method. *Radiocarbon* **30**, 171-177 (1988).

26. Bronk Ramsey C, Higham T, Bowles A, Hedges R. Improvements to the pretreatment of bone at Oxford. *46*, 155-163 (2004).

27. Williams AN. The use of summed radiocarbon probability distributions in archaeology: a review of methods. *Journal of Archaeological Science* **39**, 578-589 (2012).

28. Contreras DA, Meadows J. Summed radiocarbon calibrations as a population proxy: a critical evaluation using a realistic simulation approach. *Journal of Archaeological Science* **52**, 591-608 (2014).

29. Steel J. Radiocarbon dates as data: quantitative strategies for estimating colonization front speeds and event densities. *Journal of Archaeological Science* **37**, 2017-2030 (2010).

30. Buchanan B, Hamilton M, Edinborough K, O'Brien MJ, Collard M. A comment on Steele's (2010) "radiocarbon dates as data: quantitative strategies for estimating colonization front speeds and event densities". *Journal of Archaeological Science* **38**, 2116-2122 (2011).

31. Timpson A*, et al.* Reconstructing regional population fluctuations in the European Neolithic using radiocarbon dates: a new case-study using an improved method. *Journal of Archaeological Science* **52**, 549-557 (2014).

32. Downey SS, Bocaege E, Kerig T, Edinborough K, Shennan S. The Neolithic Demographic Transition in Europe: Correlation with Juvenility Index Supports Interpretation of the Summed Calibrated Radiocarbon Date Probability Distribution (SCDPD) as a Valid Demographic Proxy. *PLoS ONE* **9**, e105730 (2014).

33. Shennan S*, et al.* Regional population collapse followed initial agriculture booms in mid-Holocene Europe. *Nature Communications* **4:2486**, (2013).

34. Bocinsky RK, Rush J, Kintigh KW, Kohler TA. Exploration and exploitation in the macrohistory of the pre-Hispanic Pueblo Southwest. *Science Advances* **2**, e1501532 (2016).

35. Chaput MA, Gajewski K. Radiocarbon dates as estimates of ancient human population size. *Anthropocene* **15**, 3-12 (2016).

36. Pinhasi R, Fort J, Ammerman AJ. Tracing the Origin and Spread of Agriculture in Europe. *PLoS Biol* **3**, e410. (2005).

37. Andersen SH. 'Køkkenmøddinger' (shell middens) in Denmark: a survey. *Proceedings of the Prehistoric Society* **66**, 361-384 (2000).

38. Andersen SH. Shell middens ("Køkkenmøddinger") in Danish Prehistory as a reflection of the marine environment. In: *Shell Middens in Atlantic Europe* (eds Milner N, Craig OE, Bailey GN). Oxbow Books (2007).

39. Andersen SH. Shell middens (“Køkkenmøddinger”): The Danish evidence. In: *Early human impact on megamolluscs. BAR International Series, 1865* (eds Antczak AT, Cipriani R) (2008).

40. Andersen SH. Brovst. En kystboplads fra ældre stenalder. *Kuml* **1969**, 67-90 (1970).

41. Andersen SH, Rasmussen KL. Bjørnsholm. A stratified Køkkenmødding on the Central Limfjord, North Jutland. *Journal of Danish Archaeology* **10**, 59-65 (1991).

42. Bratlund B. The bone remains of mammals and birds from the Bjørnsholm shell mound; a preliminary report. *Journal of Danish Archaeology* **10**, 97–104 (1991).

43. Enghoff IB. Mesolithic Eel-Fishing at Bjørnsholm, Denmark, Spiced with Exotic Species. *Journal of Danish Archaeology* **10**, 105-118 (1991).

44. Enghoff IB. Fishing from the Stone Age Settlement Norsminde. *Journal of Danish Archaeology* **8**, 41-50 (1989).

45. Enghoff IB. Fishing in Denmark during the Mesolithic Period. In: *Man and Sea in the Mesolithic* (ed Fischer A). Oxbow Books (1995).

46. Enghoff IB, MacKenzie BR, Nielsen EE. The Danish fish fauna during the warm Atlantic period (ca. 7000-3900 BC): Forerunner of future changes? *Fisheries Research* **87**, 167-180 (2007).

47. Enghoff IB. Fishing in Denmark during the Ertebølle period. *International Journal of Osteoarchaeology* **4**, 65-96 (1994).

48. Enghoff IE. Freshwater Fishing from a Sea-Coast Settlement - the Ertebølle *locus classicus* Revisited. *Journal of Danish Archaeology* **5**, 62-76 (1986).

49. Tauber H. ^{13}C evidence for dietary habits of prehistoric man in Denmark. *Nature* **292**, 332-333 (1981).

50. Fischer A, Olsen J, Richards M, Heinemeier J, Sveinbjörnsdóttir AE, Bennike P. Coast-inland mobility and diet in the Danish Mesolithic and Neolithic: evidence from stable isotope values of humans and dogs. *Journal of Archaeological Science* **34**, 2125-2150 (2007).

51. Richards MP, Price TD, Koch E. Mesolithic and Neolithic subsistence in Denmark: New stable isotope data. *Current Anthropology* **44**, 288-U284 (2003).

52. van der Sluis LG, Reimer PJ, Ogle N. Adding hydrogen to the isotopic inventory - Combining δ13C, δ15N and δ2H stable isotope analysis for palaeodietary purposes on archaeological bone. *Archaeometry* **61**, 720-749 (2019).

53. Manning K, Colledge S, Crema E, Shennan S, Timpson A. The Cultural Evolution of Neolithic Europe. EUROEVOL Dataset 1: Sites, Phases and Radiocarbon Data. *Journal of Open Archaeology Data* **5, e2**, (2016).

54. Mellars PA. *Excavations on Oronsay: prehistoric human ecology on a small island*. Edinburgh University Press (1987).

55. Bonsall C. The 'Obanian Problem'. Coastal adaptation in the Mesolithic of western Scotland. In: *The Early Prehistory of Scotland* (eds Pollard T, Morrison A). Edinburgh Univeristy Press (1996).

56. Russel NJ, Bonsall C, Sutheland DG. The exploitation of marine molluscs in the Mesolithic of western Scotland: evidence form Ulva Cave, Inner Hebrides. In: *Man and sea in the Mesolithic: coastal settlement above and below present sea level* (ed Fischer A). Oxbow Books/Danish National Forest and Nature Agency (1995).

57. Richards MP, Schulting RJ, Hedges REM. Sharp shift in diet at onset of Neolithic. *Nature* **425**, 366 (2003).

58. Richards MP, Mellars P. Stable isotopes and the seasonality of the Oronsay middens. *Antiquity* **72**, 178-184 (1998).

59. Schulting R. Holocene environmental change and the Mesolithic-Neolithic transition in north-west Europe: revisiting two models. *Environmental Archaeology* **15**, 160-172 (2010).

60. Bonsall C, Anderson DE, Macklin MG. The Mesolithic-Neolithic transition in western Scotland and its European context. *Documenta Praehistorica XXXVI* **29**, 1–19 (2002).

61. Bonsall C, Macklin MG, Anderson DE, Payton RW. Climate change and the adoption of agriculture in northwest Europe. *European Journal of Archaeology* **5**, 9–23 (2002).

62. Schulting RJ, Richards MP. The wet, the wild and the domesticated: the Mesolithic-Neolithic transition on the west coast of Scotland. *European Journal of Archaeology* **5**, 147–189 (2002).

63. Andersen SH. Coastal adaption and marine exploitation in Late Mesolithic Denmark - with special emphasis on the Limfjord Region. In: *Man and Sea in the Mesolithic. Coastal settlement above and below present sea-level* (ed Fischer A). Oxbow Books (1995).

64. Hartz S, Heinrich D, Lübke H. Coastal Farmers - the neolithisation of northern-most Germany. In: *The Neolithisation of Denmark. 150 years of debate* (eds Fischer A, Kristiansen K). J.R. Collis (2002).

65. Andersen NH. *The Sarup enclosures. The Funnel Beaker Culture of the Sarup site including two causewayed camps compared to the contemporary settlements in the area and other European enclosures. Moesgaard, Højbjerg: Jutland Archaeological Society,*. Aarhus University Press (1997).

66. Thrane H. Danish plough-marks from the Neolithic and Bronze Age. *Journal of Danish Archaeology* **8**, 111-125 (1991).

67. Kristiansen K. De aeldste bygder; Landet äbnes. In: *Det danske landbrugs historie I. Oldtid og Middelalder. (Oldtid ved L. Hedeager og K. Kristiansen)* (ed Bjørn C) (1998).

68. Andersen ST, Rasmussen KL. Radiocarbon wiggle-dating of elm declines in northwest Denmark and their significance. *Vegetation History and Archaeobotany* **2**, 125-135 (1993).

69. Rasmussen P. Mid- to late-Holocene land-use change and lake development at Dallund Sø, Denmark: vegetation and land-use history inferred from pollen data. *The Holocene* **15**, 1116-1129 (2005).

70. Rasmussen P, Bradshaw EG. Mid- to late-Holocene land-use change and lake development at Dallund Sø, Denmark: study aims, natural and cultural setting, chronology and soil erosion history. *The Holocene* **15**, 1105-1115 (2005).

71. Rasmussen P, Olsen J. Soil erosion and land-use change during the last six millenia recorded in lake sediments from Gudme Sø, Fyn, Denmark. *Geological Survey of Denmark and Greenland Bulletin* **17**, 37-40 (2009).

72. Iversen J. Landnam i Danmarks Stenalder. (Land occupation in Denmark's Stone Age). *Danmarks Geologiske Undersøgelse II* **66**, 68 pp. (1941).

73. Iversen J. The development of Denmark's nature since the last glacial. *Danmarks Geologiske Undersøgelse* **V, 7-C.**, 126 pp. (1973).

74. Aaby B. Trees as anthropogenic indicators in regional pollen diagrams from eastern Denmark. In: *Anthropogenic indicators in pollen diagrams* (ed Behre K-E). Balkema (1986).

75. Behre K-E. The interpretation of anthropogenic indicators in pollen diagrams. *Pollen et spores* **23**, 225-245 (1981).

76. Odgaard B. The Holocene vegetation history of northern West Jutland. *Opera Botanica* **123**, 171 (1994).

77. Odgaard B. Cultural landscape development through 5500 years at Lake Skånsø, North-western Jutland as reflected in a regional pollen diagram. *Journal of Danish Archaeology* **8**, 200-210 (1989).

78. Marcott SA, Shakun JD, Clark PU, Mix AC. A Reconstruction of regional and global temperature for the past 11,300 years. *Science* **339**, 1198-1201 (2013).

79. Renssen H, Seppä H, Crosta X, Goosse H, Roche DM. Global characterization of the Holocene Thermal Maximum. *Quaternary Science Reviews* **48**, 7-19 (2012).

80. Renssen H, Seppä H, Heiri O, Roche DM, Goosse H, Fichefet T. The spatial and temporal complexity of the Holocene thermal maximum. *Nature Geoscience* **2**, 411-414 (2009).

81. Seppä H, Bjune AE, Telford RJ, Birks HJB, Veski S. Last nine-thousand years of temperature variability in Northern Europe. *Climate of the Past* **5**, 523–535 (2009).

82. Antonsson K, Seppä H. Holocene temperatures in Bohuslän, southwest Sweden: a quantitative reconstruction from fossil pollen data. *Boreas* **36**, 400-410 (2007).

83. Snowball I, Korhola A, Briffa KR, Koç N. Holocene climate dynamics in Fennoscandia and the North Atlantic. In: *Past climate variability through Europe and Africa* (eds Batterbee RW, Gasse F, Stickely CE). Kluwer Academic Publishers (2004).

84. Antonsson K, Chen D, Seppä H. Anticyclonic atmospheric circulation as an analogue for the warm and dry mid-Holocene summer climate in central Scandinavia. *Climate of the Past* **4**, 215-224 (2008).

85. Iversen J. Viscum, Hedera and Ilex as climatic indicators: a contribution to the study of the post-glacial temperature climate. *Geologiska Föreningens I Stockholm Förhandlingar* **66**, 463–483. (1944).

86. Seppä H, Hammarlund D, Antonsson K. Low-frequency and high-frequency changes in temperature and effective humidity during the Holocene in south-central Sweden: implications for atmospheric and oceanic forcings of climate. *Climate Dynamics* **25**, 285-297 (2005).

87. Brown KJ, Seppä H, Schoups G, Fausto R, Rasmussen P, Birks HJB. A spatio-temporal reconstruction of Holocene temperature change in southern Scandinavia. *The Holocene* **22**, 165-177 (2012).

88. Knudsen KL, Conradsen K, Heier-Nielsen S, Seidenkrantz MS. Quaternary palaeoceanography and palaeogeography in Northern Denmark: a review of results from the Skagen cores. *Bulletin of the Geological Society of Denmark* **43**, 22-33 (1996).

89. Vork KA, Thomsen E. Lusitanian/Mediterranean ostracods in the Holocene of Denmark: Implications for the interpretation of winter temperatures during the postglacial temperature maximum. *The Holocene* **6**, 423-432 (1996).

90. Sommer RS, Persson A, Wieseke N, Fritz U. Holocene recolonization and extinction of the pond turtle, *Emys orbicularis* (L., 1758), in Europe. *Quaternary Science Reviews* **26**, 3099-3107 (2007).

91. Ljungar L. First subfossil finds of the Aesculapian snake, *Elaphe longissima* (Laur.) (Colubridae) from a Mesolithic settlement in Denmark. *Amphibia-Reptilia* **16**, 93-94 (1994).

92. Warden L*, et al.* Climate-induced human demographic and cultural change in northern Europe during the mid-Holocene. *Scientific Reports* **7:15251**, (2017).

93. Davis BAS, Brewer S, Stevenson AC, Guiot J, Contributors D. The temperature of Europe during the Holocene reconstructed from pollen data. *Quaternary Science Reviews* **22**, 1701–1716 (2003).

94. Tallavaara M, Seppä H. Did the mid-Holocene environmental changes cause the boom and bust of hunter-gatherer population size in eastern Fennoscandia? *The Holocene* **22**, 215–225 (2011).

95. Kotthoff U*, et al.* Reconstructing Holocene temperature and salinity variations in the western Baltic Sea region: a multi-proxy comparison from the Little Belt (IODP Expedition 347, Site M0059). *Biogeosciences* **14**, 5607-5632 (2017).

96. Tierney JE. Biomarker-Based Inferences of Past Climate: The TEX86 Paleotemperature Proxy. *Treatise on Geochemistry* **2nd Edition**, 379-393 (2014).

97. Lambeck K, Smither C, Johnston P. Sea-level change, glacial rebound and mantle viscosity for northern Europe. *Geophysical Journal International* **134**, 102-144 (1998).

98. Björck S. A Review of the History of the Baltic Sea. 13.0-8.0 ka BP. *Quaternary International* **27**, 19-40 (1995).

99. Andrén T, Björck S, Andrén E, Conley D, Zillén L, Anjar J. The development of the Baltic Sea during the last 130 ka. In: *The Baltic Sea Basin* (eds Harff J, Björck S, Hoth P). Springer-Verlag (2011).

100. Mertz EL. Late and post-glacial height changes in Denmark (In Danish). *Danish Geological Survey (DGU) 2* **41**, 50 (1924).

101. Yu S-Y. Centennial-scale cycles in middle Holocene sea level along the southeastern Swedish Baltic coast. *GSA Bulletin* **114**, 1404-1409 (2003).

102. Yu S-Y, Berglund BE. A dinoflagellate cyst record of Holocene climate and hydrological changes along the southeastern Swedish Baltic coast. *Quaternary Research* **67**, 215-224 (2007).

103. Petersen K-S. The Holocene marine transgression and its molluscan fauna in the Skagerrak-Limfjord region, Denmark. *Special Publications International Association of Sedimentologists* **5**, 497-503 (1981).

104. Christensen C. The Littorina transgressions in Denmark. In: *Man and sea in the Mesolithic. Coastal settlement above and below present sea-level* (ed Fischer A). Oxbow Books (1995).

105. Pedersen JBP, Svinth S, Bartholdy J. Holocene evolution of a drowned melt-water valley in the Danish Wadden Sea. *Quaternary Research* **72**, 68-79 (2009).

106. Clemmensen LB, Murray AS, Nielsen L. Quantitative constraints on the sea-level fall that terminated the Littorina Sea Stage, southern Scandinavia. *Quaternary Science Reviews* **40**, 54-63 (2012).

107. Berglund BE*, et al.* Early Holocene history of the Baltic Sea, as reflected in coastal sediments in Blekinge, southeastern Sweden. *Quaternary International* **130**, 111-139 (2005).

108. Christensen C. Coastal settlement and sea level change in the Stone Age. In: *Denmarks Hunting Stone Age – status and perspectives* (eds Jensen OL, Sørensen SA, Hansen KM). Hørsholm Egns Museum (2001).

109. Yu S-Y, Berglund BE, Sandgren P, Lambeck K. Evidence for a rapid sea-level rise 7600 yr ago. *Geology* **35**, 891-894 (2007).

110. Peltier WR. On esutatic sea level history: Last Glacial Maximum to Holocene. *Quaternary Science Reviews* **21**, 377-396 (2002).

111. Gregersen S, Voss P. Irregularities in Scandinavian postglacial uplift/subsidence in time scales tens, hundreds, thousands of years. *Journal of Geodynamics* **50**, 27–31 (2010).

112. Jensen S, Spärck R. Bløddyr II. Saltvandsmuslinger. *Danmarks Fauna* **40**, 208 (1934).

113. Yonge CM. *Oysters*. Collins (1960).

114. Jeffrey S, Mantoura R, Wright S. *Phytoplankton Pigments in Oceanography: Guidelines to Modern Methods.* UNESCO publishing (1997).

115. Fischer A, Kristiansen K. *The Neolithisation of Denmark. 150 years of debate*. J.R.Collis (2002).

116. Burman J, Schmitz B. Periwinkle (*Littorina littorea*) intrashell delta $\delta^{18}O$ and delta $\delta^{13}C$ records from the mid-Holocene Limfjord region, Denmark: a new high-resolution palaeoenvironmental proxy approach. *The Holocene* **15**, 567-575 (2005).

117. Hofmeister R, Bolding K, Burchard H. Managing benthic ecosystems in relation to physical forcing and environmental constraints. MaBenE Deliverable D1.1. Report about Limfjord model setup and results.) (2006).

118. Muus BJ. *The fauna of Danish estuaries and lagoons. Meddelelser fra Danmarks Fiskeri- og Havundersøgelser* (1967).

119. Conley DJ, Kaas H, Mohlenberg F, Rasmussen B, Windolf J. Characteristics of Danish estuaries. *Estuaries* **23**, 820-837 (2000).

120. Markager S, Stedmon CA, Søndergaard M. Seasonal dynamics and conservative mixing of dissolved organic matter in the temperate eutrophic estuary Horsens Fjord. *Estuarine, Coastal and Shelf Science* **92**, 376-388 (2011).

121. Sorgenfrei T. Molluscan assemblages from the marine middle Miocene of South Jutland and their environment. *Danmarks Geologiske Undersøgelse, II* **79**, 356-503 (1958).

122. Petersen K-S. Late Quaternary environmental changes recorded in the Danish marine molluscan faunas. *Geological Survey of Denmark and Greenland Bulletin* **3**, 268 (2004).

123. Ellis BF, Messina A. *Catalogue of Foraminifera (with supplements, including 2009)*. American Museum of Natural History and Micropaleontology Press (1949).

124. Bennike O, Rasmussen P, Aaris-Sørensen K. The harp seal (*Phoca groenlandica* Erxleben) in Denmark, southern Scandinavia, during the Holocene. *Boreas* **37**, 263–272 (2008).

125. Nikulina EA, Schmölcke U. First archaeogenetic results verify the mid-Holocene occurrence of Dalmatian pelican Pelecanus crispus far out of present range. *Journal of Avian Biology* **46**, 344–351 (2015).

126. Erbs-Hansen DR, Knudsen K-L, Gary AC, Gyllencreutz R, Jansen E. Holocene climatic development in Skagerrak, eastern North Atlantic: Foraminiferal and stable isotopic evidence. *The Holocene* **22**, 301–312 (2012).

127. Rasmussen P, Petersen KS, Ryves DB. Environmental change in Danish marine waters during the Roman Warm Period inferred from mollusc data. *Geological Survey of Denmark and Greenland Bulletin* **13**, 21-24 (2007).

128. Renberg I. The HON-Kajak sediment corer. *Journal of Paleolimnology* **6**, 167-170 (1991).

129. Pedersen L. 7000 years of fishing: stationary fishing structures in the Mesolithic. In: *Man and Sea in the Mesolithic* (ed Fischer A). Oxbow Books (1995).

130. Pickard C, Bonsall C. Late Mesolithic coastal fishing practices: the evidence from Tybrind Vig, Denmark. In: *On the Road. Studies in Honour of Lars Larsson. Acta Archaeologica Lundensia in 4°, No. 26* (eds Hårdh B, Jennbert K, Olausson D). Almqvist and Wiksell (2007).
