# Supplementary material for: Marine resource abundance drove pre-agricultural population increase in Stone Age Scandinavia
Source: Nat Commun. 2020 Apr 24;11:2006. doi: 10.1038/s41467-020-15621-1 (PMC7181652; doi:10.1038/s41467-020-15621-1)
Supplement: Supplementary file 2 — Description of Additional Supplementary Information [file 41467_2020_15621_MOESM2_ESM.pdf]

Description of Additional Supplementary Files

File Name: Supplementary Data 1
Description: Holocene stable isotope data obtained from bone collagen from human and dog remains from Denmark.
